# The SMARCA4 subunit of the SWI/SNF complex prevents genome instability at G quadruplexes

**DOI:** 10.1186/s13059-026-04080-4

**Published:** 2026-04-20

**Authors:** Alison Harrod, Hugang Feng, Nagham Ghaddar, Federica Schiavoni, Karen A. Lane, Lillian Wu, Pedro Zuazua-Villar, Zuzanna Kozik, Felix M. Dobbs, Patrick van Eijk, Gene Ching Chiek Koh, Navita Somaiah, Jyoti Choudhary, Serena Nik-Zainal, Simon H. Reed, Jessica A. Downs

**Affiliations:** 1 Division of Cell and Molecular Biology, The Institute of Cancer Research, London, UK; 2 Present address: The Francis Crick Institute, London, UK; 3 Division of Cancer and Genetics, School of Medicine, Cardiff University, Cardiff, UK; 4 Broken String Biosciences, BioData Innovation Centre, Wellcome Genome Campus, Unit AB303, Level 3, Cambridge, Hinxton UK; 5 Department of Genomic Medicine, School of Clinical Medicine, University of Cambridge, Cambridge, UK; 6 Sir Jeffrey Cheah Sunway Medical School, Faculty of Medical and Life Sciences, Sunway University, Sunway City, Malaysia; 7 Division of Radiotherapy and Imaging, The Institute of Cancer Research, London, UK

**Keywords:** SMARCA4, BRG1, SWI/SNF, Genome instability, G quadruplex, Pyridostatin

## Abstract

**Background:**

G-quadruplex (G4) structures are secondary structures that can form in guanine-rich single stranded DNA sequences. These play important roles in biological processes such as regulation of gene expression but can also pose challenges to DNA replication and lead to genome instability. The SMARCA4 (BRG1) subunit of the SWI/SNF chromatin remodelling complexes has been identified as a G4 binding protein, and evidence suggests that this interaction can promote SWI/SNF-dependent gene expression. SMARCA4 is frequently misregulated in cancer, where genome instability is common, but whether there is an impact of SMARCA4 on G4 stability was not known.

**Results:**

Here, we show that SMARCA4 prevents genome instability at G4s. Mapping unrepaired DNA breaks reveals that these preferentially co-localise with G4 forming structures in SMARCA4-deficient cells. Moreover, using whole genome sequencing approaches, we find that misrepair events in SMARCA4-deficient cells are more likely to map to G4 forming sequences. Consistent with this, SMARCA4-deficient cells show sensitivity to the G4 ligand pyridostatin and defective pyridostatin-induced DNA damage responses. Notably, analysis of cancer patient data shows that SMARCA4-deficient samples have an increased proportion of G4-associated mutations when compared with SMARCA4-proficient samples.

**Conclusions:**

These findings suggest that SMARCA4 plays a crucial role in maintaining stability at G4 motifs. This insight provides valuable information about the functional significance of G4 structures and their interaction with SMARCA4, particularly in the context of cancer.

**Supplementary Information:**

The online version contains supplementary material available at 10.1186/s13059-026-04080-4.

## Background

G quadruplexes (G4s) are secondary structures that can form in guanine-rich stretches of nucleic acids in cells [1–3]. These four-stranded structures provide important biological functions, such as regulating telomere biology and gene expression [4]. G4 sequences are enriched in regulatory regions of the genome, suggesting a functional role [5, 6]. There is evidence that they impact on gene expression by creating transcription factor binding sites [7], functioning as a binding site for chromatin remodellers [8], regulating RNA polymerase II binding or pausing [9, 10], and influencing three-dimensional chromatin organisation including topologically-associating domains (TADs) and enhancer-promoter interactions [11].

While these structures play an important physiological role, G4s can create challenges for the ability of cells to maintain genome stability [12]. G4s can act as barriers to the progression of both RNA and DNA polymerases, thus leading to difficulties during transcription and replication [13–15]. These difficulties can lead to the generation of persistent single stranded DNA and DNA breaks, both of which can lead to mutagenic outcomes. This effect of G4s on genome instability is exacerbated by G4-binding ligands such as pyridostatin (PDS), which leads to increased DNA double strand break formation [16]. Cells with defects in pathways important for replication stress, DNA repair, and G4 resolution show increased PDS sensitivity [17, 18].

Genome instability at G4s is apparent in cancer samples at a rate greater than expected by chance [19–22]. Translocation breakpoints in cancer samples are overrepresented at G4s, and correlation analysis showed a relationship with dysregulated cell cycle and DNA repair, including TP53 [20]. However, whether and how these mutational profiles are influenced by loss of pathways that resolve G4s or respond to G4-induced DNA damage are relatively unexplored.

SMARCA4 (or BRG1) is a catalytic subunit of mammalian SWI/SNF chromatin remodelling complexes that contribute to gene expression regulation through activity at promoters and enhancers [23–25]. Notably, SWI/SNF subunits are found in G4-interactomes (*e.g.* [26]), and SMARCA4 was identified as a G4 binding protein that maps to G4 sequences in cells [27]. SMARCA4 is frequently dysregulated in cancer [25, 28–30], and SWI/SNF complexes contribute to genome stability through DNA damage responses [28]. We therefore considered the possibility that SMARCA4 might play a protective role at G4 sequences.

Here, we show that SMARCA4 prevents genome instability at G4s. In SMARCA4-deficient cells, we find that unrepaired DNA breaks preferentially co-localise with G4 forming sequences. In addition, when mutations are analysed by whole genome sequencing, we find these are more likely to map to G4 forming sequences in the absence of SMARCA4. SMARCA4-deficient cells show sensitivity to the G4 ligand pyridostatin (PDS) and show impaired DNA damage responses after PDS treatment. Importantly, an increased propensity for mutations to be associated with G4 sequences was found in patients with *SMARCA4*-mutant tumours when compared with *SMARCA4* wild-type patient groups. These findings suggest that SMARCA4 plays a crucial role in maintaining stability at G4 motifs. This insight provides valuable information about the functional significance of G4 structures and their interaction with SMARCA4, particularly in the context of cancer.

## Results

### SMARCA4 binds to G quadruplex sequences

To understand more about the relationship between SMARCA4 and G4 structures in the genome, we mapped SMARCA4 in RPE1 cells using Cleavage Under Targets & Release Using Nuclease (CUT&RUN). We then analysed the relationship between SMARCA4 binding sites and G4-forming sequences using three G4 databases. Predicted G Quadruplex-forming Sequences (PQS) uses sequence motifs to predict G4-forming sequences and therefore captures the widest number of potential G4 overlaps and avoids bias. However, many of these sites will not form G4s in cells, so we also used Quadron [31], which uses machine learning to estimate the likelihood of G4 formation and uses this to filter G4 sites, yielding a smaller but potentially more physiological dataset. Finally, we made use of a dataset in which an antibody that recognises these structures, BG4, was used to map G4s in HEK293T cells [32]. While this is the most biologically relevant dataset, cell line specific differences will be present and likely bias the results. In addition, the relatively low number of mapped G4s limited the analyses that were possible with this dataset. Therefore, by using all three datasets (wherever possible), we minimise the limitations inherent to any single choice.

**Fig. 1:**
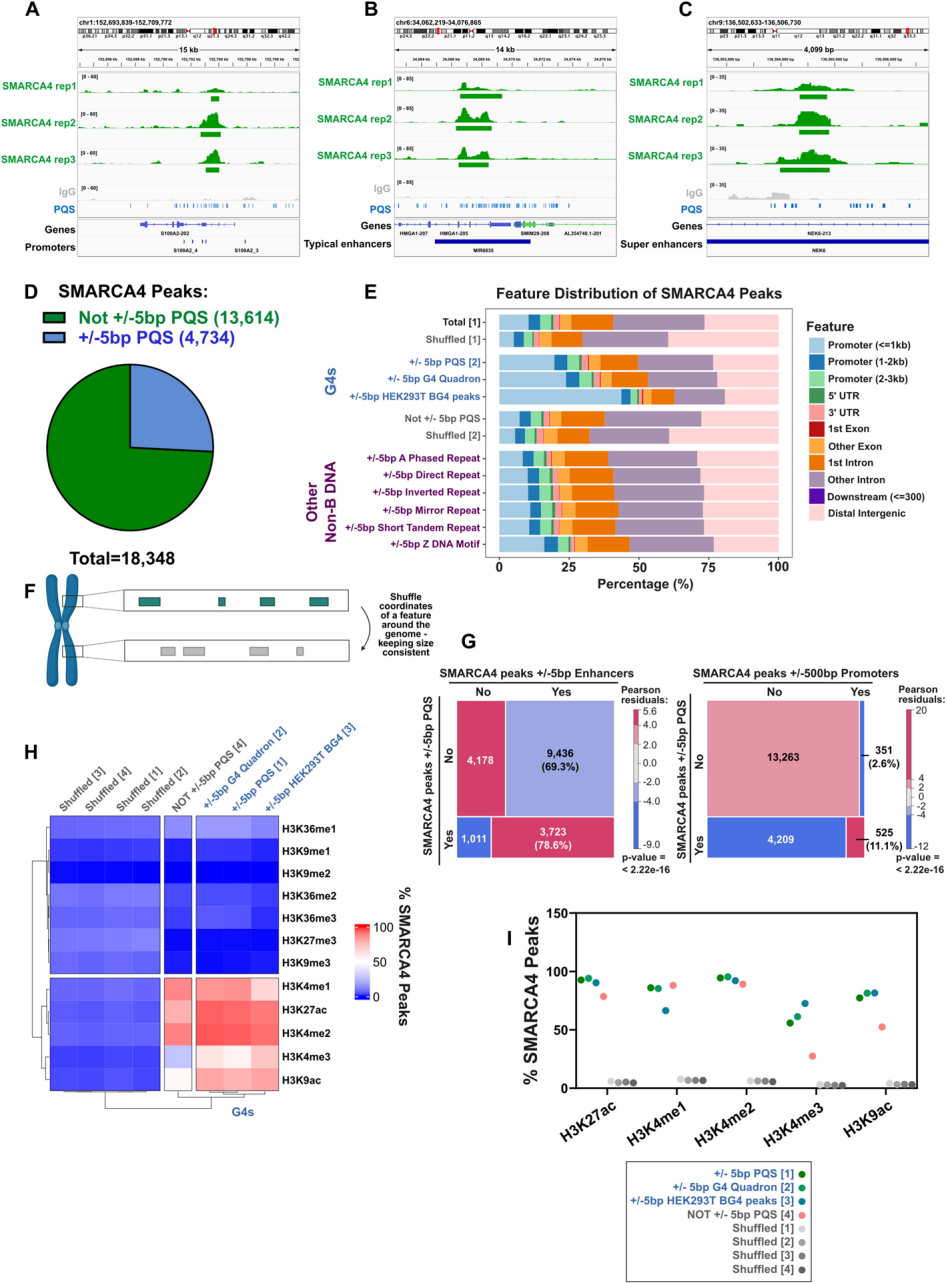
SMARCA4 binds at predicted G4 sites genome wide. **A-C** Representative genome tracks displaying coverage of reads from 3 independent biological replicates of SMARCA4 CUT&RUN in the RPE1 parental cells (green), and 1 representative replicate of the IgG antibody negative control. Predicted G4 sequences (PQS) are highlighted in blue, and annotations for genes and either promoters (**A**), typical enhancers (**B**) or super enhancers (**C**) are shown. **D** Pie chart representing the proportion of SMARCA4 consensus peaks (significant q-value < 0.01, in at least 2 replicates) that are within a 5 bp window of a PQS. **E** Stacked colour bar chart representing the genomic distribution of significant SMARCA4 peaks, categorised by feature. **F** Schematic explaining how coordinates are shuffled for the shuffled controls. **G** Mosaic plots of observed frequencies of cooccurrence of SMARCA4 consensus peaks with PQS (within 5 bp window) and either enhancers (within 5 bp window, left) or promoters (within 500 bp window, right). Numbers of SMARCA4 peaks in each subset are annotated, with percentages of peaks in each row that cooccur with enhancers or promoters, respectively. Tile sizes are proportional to the observed frequencies. Shading indicates Pearson residuals from chi-square test; red indicates positive residual and blue indicates negative residual. Overall p-value indicates evidence for significant difference from expected values. **H** Heatmap representing the percentage of SMARCA4 peaks within 5 bp window of significant histone PTM peaks, with hierarchical clustering. **I** Dot plot representing the percentage of SMARCA4 peaks within 5 bp window of histone PTM peaks, as in (**H**), but focused on active histone marks. For **E**, **H** and **I**, shuffled controls are where the coordinates of peak groups (specified in square brackets) are shuffled around the genome. Figure **F** was generated using BioRender

SMARCA4 binding sites overlapped with G4s at a variety of locations, including promoters, enhancers and super enhancers (Fig. 1A-C and Additional file 1: Fig. S1A-D). When peaks identified in at least two of three biological replicates were analysed (Additional file 1: Fig. S1E), we found that approximately 25% of SMARCA4 binding sites are located within 5 bp of a PQS (Fig. 1D). The overlap between SMARCA4 peaks and G4s was slightly lower when we used G4 sites predicted by Quadron or mapped with the BG4 antibody (Additional file 1: Fig. S1B, C), reflecting lower numbers in these datasets (Additional file 1: Fig. S1D, E), but nevertheless represented a substantial fraction of SMARCA4 binding sites, consistent with a previous report [27].

When the distribution of total SMARCA4 binding sites was analysed, the population associated with G4 sequences shows a shift towards promoters when compared with total SMARCA4 binding sites (Fig. 1E and Additional file 1: Fig. S1F). This was not evident when SMARCA4 peaks associated with other non-B form DNA structures, such as inverted repeats or short tandem repeats, was similarly analysed (Fig. 1E and Additional file 1: Fig. S1F). However, unlike SMARCA4, these structures are less often found in open chromatin (Additional file 1: Fig. S1G-I), raising the possibility that the relationship between SMARCA4 and G4s at promoters simply reflects their abundance at regions of open chromatin. Therefore, as an additional control, we randomly shuffled the sequences and intersected them (Fig. 1F) and found that the increased percentage of promoter-associated SMARCA4 peaks near G4s is greater than expected by chance (Fig. 1E and Additional file 1: Fig. S1F). To interrogate these numbers more rigorously, we performed Pearson chi-square analysis and plotted the data as mosaic plots that show the deviation from the expected frequency (see Additional file 1: Fig. S1J for an explanation of mosaic plots). In this analysis, when SMARCA4 peaks intersecting with G4s identified using PQS or Quadron are analysed, the association with promoters and enhancers is significantly increased relative to the total population of SMARCA4 peaks (Fig. 1G and Additional file 1: Fig. S1K). Together, these data suggest that the relationship between SMARCA4 and G4s is dominated by the population of SMARCA4 in regulatory elements.

Consistent with this, when SMARCA4 peaks are divided based on proximity to G4s (using either PQS, Quadron, or BG4 mapping versus those not near PQS G4s), the G4-associated peaks are in chromatin containing histone marks H3K4me3 and H3K9ac, which are enriched at promoters and actively transcribed genes, to a much greater degree than peaks not near G4s or when compared with the shuffled controls (Fig. 1H, I).

### Loss of SMARCA4 leads to increased proportion of double strand breaks (DSBs) at G4-forming sequences

G4 structures have been shown to act as an impediment to replication, leading to the generation of DNA double strand breaks (DSBs) and genome instability [14, 16], and SMARCA4-deficient cells have been shown to display signs of replication stress (for example, [33]). Moreover, SMARCA4 and other SWI/SNF subunits are enriched at sites of stalled replication forks [34]. This raises the interesting possibility that SMARCA4 contributes to replication fork stability at G4s. If so, SMARCA4 deficiency might lead to increased DSBs at G4s in otherwise unperturbed cells.

**Fig. 2:**
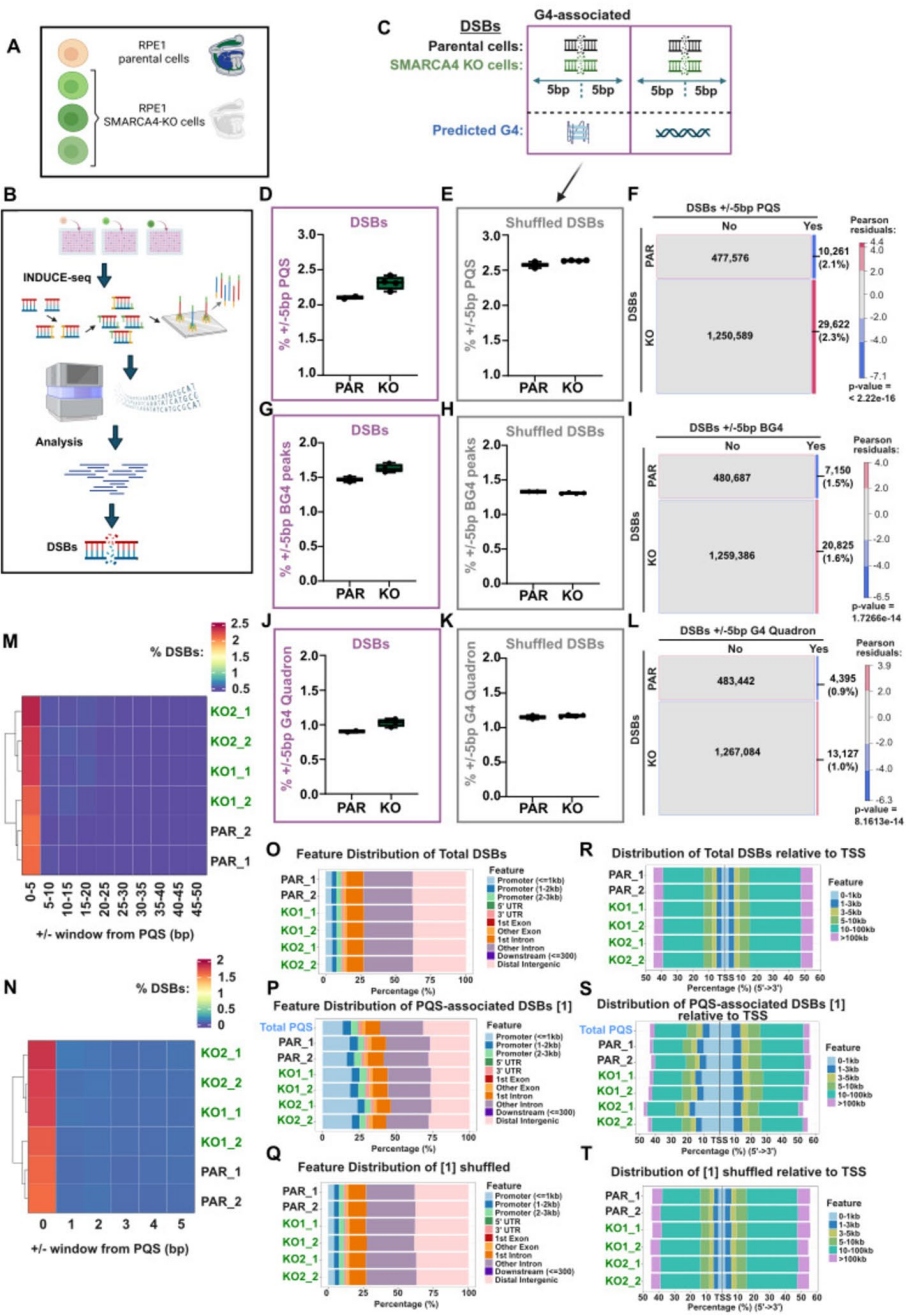
DSBs are more likely to occur near predicted G4s in the absence of SMARCA4. **A** Schematic representing *SMARCA4* KO clones used in the study. **B** Simplified workflow of DSB mapping by INDUCE-seq. **C** Schematic representing DSBs in parental or *SMARCA4* KO cells within a 5 bp window of a predicted G4, or not. **D-E** Box and whisker plot indicating percentage of DSBs (**D**) and shuffled DSBs (**E**) within a 5 bp window of PQS sites, for parental (PAR) and *SMARCA4* KO clones (KO). **F** Mosaic plot of observed frequencies of cooccurrence of DSBs (replicates and KO clones combined) with PQS sites (within 5 bp window). **G**,** H** Box and whisker plot for the percentage of DSBs (**G**) and shuffled DSBs (**H**) within 5 bp window of significant BG4 peaks. **I** Mosaic plot for cooccurrence of DSBs with significant BG4 peaks. **J**,** K** Box and whisker plot for the percentage of DSBs (**J**) and shuffled DSBs (**K**) within 5 bp window of G4 Quadron sites. **L** Mosaic plot for cooccurrence of DSBs with G4 Quadron sites. For **F**, **I** and **L**, numbers in each subset are annotated, with percentages of DSBs in each row that cooccur with G4s. Tile sizes are proportional to observed frequencies. Red indicates positive and blue indicates negative Pearson residuals from chi-square test. P-value indicates evidence for significant difference from expected values. **M**,** N** Hierarchical heatmaps representing the percentage of DSBs that are within a window of PQS sites, either shown in 5 bp bins from 0–50 bp (M) or in 1 bp bins from 0–5 bp (N). **O-T** Stacked colour bar charts representing genomic distribution of total DSBs (**O**, **R**), DSBs within a 5 bp window of PQS (**P**, **S**) or a shuffled control (of DSB groups specified in square brackets) (**Q**, **T**), categorised by feature (**O**, **P**, **Q**) or relative distance to TSS (**R**, **S**, **T**). In **P** and **S**, distribution of total PQS sites is plotted for comparison. Figures **A**, **B** and **C** were generated using BioRender

To test this, we mapped the locations of unrepaired DNA DSBs in *SMARCA4* KO clones (Fig. 2A and Additional file 1: Fig. S2A) and the isogenic parental RPE1 control. We did this using INDUCE-seq (Fig. 2B) [35], which enables unbiased and quantitative mapping of DNA DSBs at precise genomic locations. We intersected the presence of unrepaired DNA breaks in our INDUCE-seq data with the predicted and mapped G4 datasets (Fig. 2C and Additional file 1: Fig. S2B) and found that there is an increased propensity for the DSBs to be located at a G4 forming sequence (using a window of ± 5 bp; hereafter ‘G4-associated’) in the KO cells relative to the parental cells (Fig. 2D-L and Additional file 1: Fig. S2B). While modest, this increased association of DSBs with G4s in the *SMARCA4* KO lines was significant when compared with the parental cells using Pearson chi-square analysis, regardless of the G4 dataset used in the analysis (Fig. 2F, I, L) and was no longer apparent when a shuffled control was used (Fig. 2E, H, K). Moreover, intersecting the DSB locations with other non-B form DNA structures showed no similar differences between the parental and *SMARCA4* KO cells (Additional file 1: Fig. S2C-T).

These data suggest that SMARCA4 plays a protective role in the vicinity of G4s. In our analyses, we used a window of 5 bp, raising the question of how far the protective effect of SMARCA4 extends from a G4. To look at this, we plotted the frequency of DSBs within a defined distance of a G4 in 5 bp windows. Using this analysis, an increase in DSBs near G4s is apparent in all cell lines and again shows an increase in the *SMARCA4* KO lines, but importantly, there is no noticeable enrichment of DSBs beyond 5 bp (Fig. 2M and Additional file 1: Fig. S2U). We therefore repeated the analysis using 1 bp windows, and found that, strikingly, the enrichment is driven by DSBs that intersect (0 bp) with G4 sequences (Fig. 2N and Additional file 1: Fig. S2V). These data suggest that the DSBs in both SMARCA4-proficient and -deficient cells arise within G4s, and that these either arise more frequently or are repaired less efficiently in the *SMARCA4* KO cells.

We then looked at the features associated with the mapped DSBs and found that DSBs near G4s are more likely to be promoter-proximal than those in the total DSB population or when compared with a shuffled control (Fig. 2O-Q and Additional file 1: Fig. S3A). In addition, the G4-associated DSBs are more likely to be in open chromatin (Additional file 1: Fig. S3B, C), associated with promoters or enhancers (Additional file 1: Fig. S3D, E), and associated with promoter enriched histone PTMs such as H3K27ac and H3K4me3 (Additional file 1: Fig. S3F, G). This relationship reflects the enrichment of G4s within promoters and nuclease hypersensitive sites [36], and these patterns were not substantially different in the *SMARCA4* KO cells. When the distribution of DSBs relative to transcription start sites (TSS) was analysed, we found that, as expected, G4-associated breaks are more likely to be near TSS than those DSBs in the total population (Fig. 2R-T and Additional file 1: Fig. S3H).

Because both G4s and SMARCA4 are enriched at promoters and enhancers, it is possible that the increased association of DSBs with G4s in the SMARCA4-deficient cells is in fact driven by association with other features, such as open chromatin or histone marks associated with promoters or enhancers (Additional file 1: Fig. S3C-G). To test this possibility, we removed DSBs located in open chromatin, at regulatory elements (promoters or enhancers), or associated with active histone PTMs and tested whether there was still a relationship between G4s and DSBs in the *SMARCA4* KO cells. Notably, in all three cases, and using both PQS and Quadron databases, *SMARCA4* KO cells had significant enrichment of G4-associated DSBs even after excluding these potentially confounding features (Additional file 1: Fig. S3I-N).

Together these data show that in the absence of SMARCA4, unrepaired DNA breaks in unperturbed cells are more likely to be located directly within a G4 forming sequence when compared with SMARCA4-proficient cells, suggesting that SMARCA4 plays a protective role at G4 structures.

### Increased frequency of genome instability at G4 sequences in SMARCA4-deficient cells

While these are modest differences, DSBs are challenging lesions and their repair, or misrepair, can lead to genetic alterations including base changes, insertions or deletions, and larger structural variants such as translocations [19–22]. Our DSB mapping data suggests that DSBs near G4s either arise with greater frequency or persist in *SMARCA4* KO cells and therefore might be particularly vulnerable to instability in the absence of SMARCA4.

**Fig. 3:**
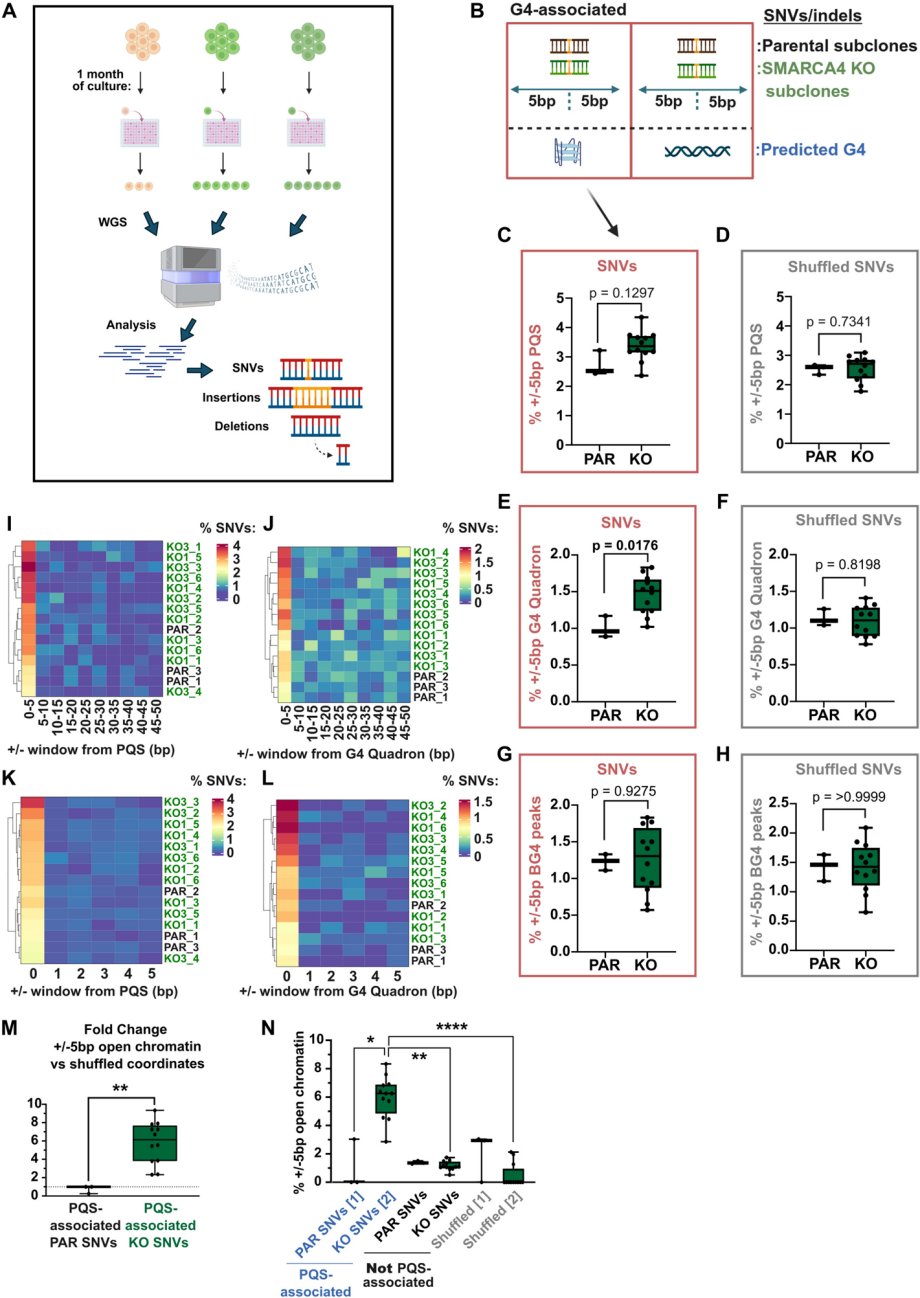
SNVs are more likely to occur near predicted G4s in the absence of SMARCA4. **A** Simplified workflow indicating whole genome sequencing following single cell cloning and cell culture. **B** Schematic representing SNVs in parental or *SMARCA4* KO cells within a 5 bp window of a predicted G4, or not. **C-H** Box and whisker plots indicating percentage of SNVs within a 5 bp window of predicted G4 sites, with clones plotted as individual points. Box and whisker plots represent the percentage of SNVs (**C**, **E**, **G**) or the shuffled coordinates of SNVs for each clone (**D**, **F**, **H**) within 5 bp windows of either PQS (**C**, **D**), G4-Quadron sites (**E**, **F**) or significant BG4 peaks (**G**, **H**). **I-L** Heatmaps representing the percentage of SNVs that are within a window of PQS sites (**I**, **K**) or G4 Quadron sites (**J**, **L**), either shown in 5 bp bins from 0–50 bp (**I**, **J**) or in 1 bp bins from 0–5 bp (**K**, **L**). **M** Box and whisker plot representing the fold change of PQS-associated SNVs that are within open chromatin (defined as within a 5 bp window of ATAC-seq peaks, which were identified in both replicates) versus the shuffled control, where coordinates of PQS-associated SNVs are shuffled around the genome. This is plotted separately for parental RPE1 and *SMARCA4* KO PQS-associated SNVs. A pseudo-count of 1 was added to both values to calculate the fold change, to prevent division by 0. **N** Box and whisker plot representing the percentage of SNVs in open chromatin (defined as in **M**). Statistical significance was assessed using a Kruskal–Wallis test with Dunn’s multiple comparisons test. For **M** and **N**, SNVs are defined either as PQS-associated, Not PQS-associated, or shuffled controls (of groups specified in square brackets). For **C-H** and **M**, statistical significance was assessed using a Mann Whitney test. For **C-H** and **M–N**, error bars indicate min to max values, and all replicates are shown as dots. Figures **A** and** B** were generated using BioRender

To test this, we performed whole genome sequencing on daughter subclones derived from two *SMARCA4* KO cell line clones and the parental RPE1 control (Additional file 1: Fig. S4A) that were cultured under unperturbed conditions for one month to allow for accumulation of genetic alterations (Fig. 3A). Western blotting was used to ensure that SMARCA4 expression was unaltered during the long-term culturing and clonal expansion (Additional file 1: Fig. S4B-D). We mapped the location of single nucleotide variations (SNVs) or insertion/deletion (indel) mutational events in order to intersect them with the G4 datasets (Fig. 3B). While there were sufficient SNVs for this analysis, the numbers of indels were too low and were not analysed further (Additional file 1: Fig. S4E).

Similar to the DSB data, we found that the percentage of G4-associated SNVs were higher in the *SMARCA4* KO cells when compared with the parental, and, while only significant when using the Quadron dataset, these differences were no longer apparent when the shuffled controls were performed (Fig. 3C-H). We further tested the window of effect relative to the G4, and found, again similar to the DSB mapping, that the G4-associated SNVs directly overlap with G4 sequences (Fig. 3I-L). Moreover, hierarchical clustering of these data highlights the increased frequency of SNVs in the *SMARCA4* KO cells compared with the parental lines (Fig. 3I-L).

As above, we analysed the feature distribution associated with SNVs—either total SNVs (Additional file 1: Fig. S4F, G) or when split into SNVs near PQS or not (Additional file 1: Fig. S4H, I). We find the G4-associated SNVs are enriched at promoter regions and TSSs when compared with either the total SNVs, SNVs not near G4s, or shuffled controls (Additional file 1: Fig. S4F-I). These patterns are very similar to the mapped DSBs and suggest that the G4-associated DSBs could contribute to the increased SNVs in these regions. Notably, using analyses of these features, there is no substantial difference in the genomic features of G4-associated SNVs between parental and *SMARCA4* KO cells.

However, when we looked at the association of chromatin accessibility with SNVs, we found that there is a greater association of G4-associated SNVs with open chromatin in the *SMARCA4* KO cells when compared with the parental, which is not apparent in the population of SNVs that are not near G4s (Fig. 3M, N). Consistent with this, we find that G4-associated SNVs are more likely to be associated with the active histone PTMs H3K27ac, H3K4me2/3, and H3K9ac in the *SMARCA4* KO cells (Additional file 1: Fig. S4J). This is not the case for SNVs that are not near G4s, and the association is greater than expected by chance (Additional file 1: Fig. S4J).

We compared the SNV patterns to other non-B form DNA structures, such as inverted or direct repeats. Unlike the analysis with predicted and mapped G4s, we found no significant differences between the parental and *SMARCA4* KO cell lines in the association of SNVs with most of these structures when compared with the shuffled controls (Additional file 1: Fig. S5A-L).

To again test whether the differential SNV associations are driven by features other than G4s, we first plotted SNVs associated with total promoters, enhancers, or open chromatin in the parental and *SMARCA4* KO cells and found no significant differences (Additional file 1: Fig. S5M-O). Next, we excluded the SNVs found in open chromatin, at regulatory elements (promoters or enhancers), or associated with active histone PTMs and reassessed the association between the remaining SNVs and G4s. Even after these exclusions, we find SNVs are more strongly associated with G4s in the *SMARCA4* KO cells (Additional file 1: Fig. S5P-U).

Together, these data show that mutations accumulate more frequently at G4 sequences in the absence of SMARCA4, and they suggest that this is driven in part by mutations arising in accessible, gene regulatory regions of chromatin.

### Sites where SMARCA4 was bound are disproportionately prone to DSB formation

**Fig. 4:**
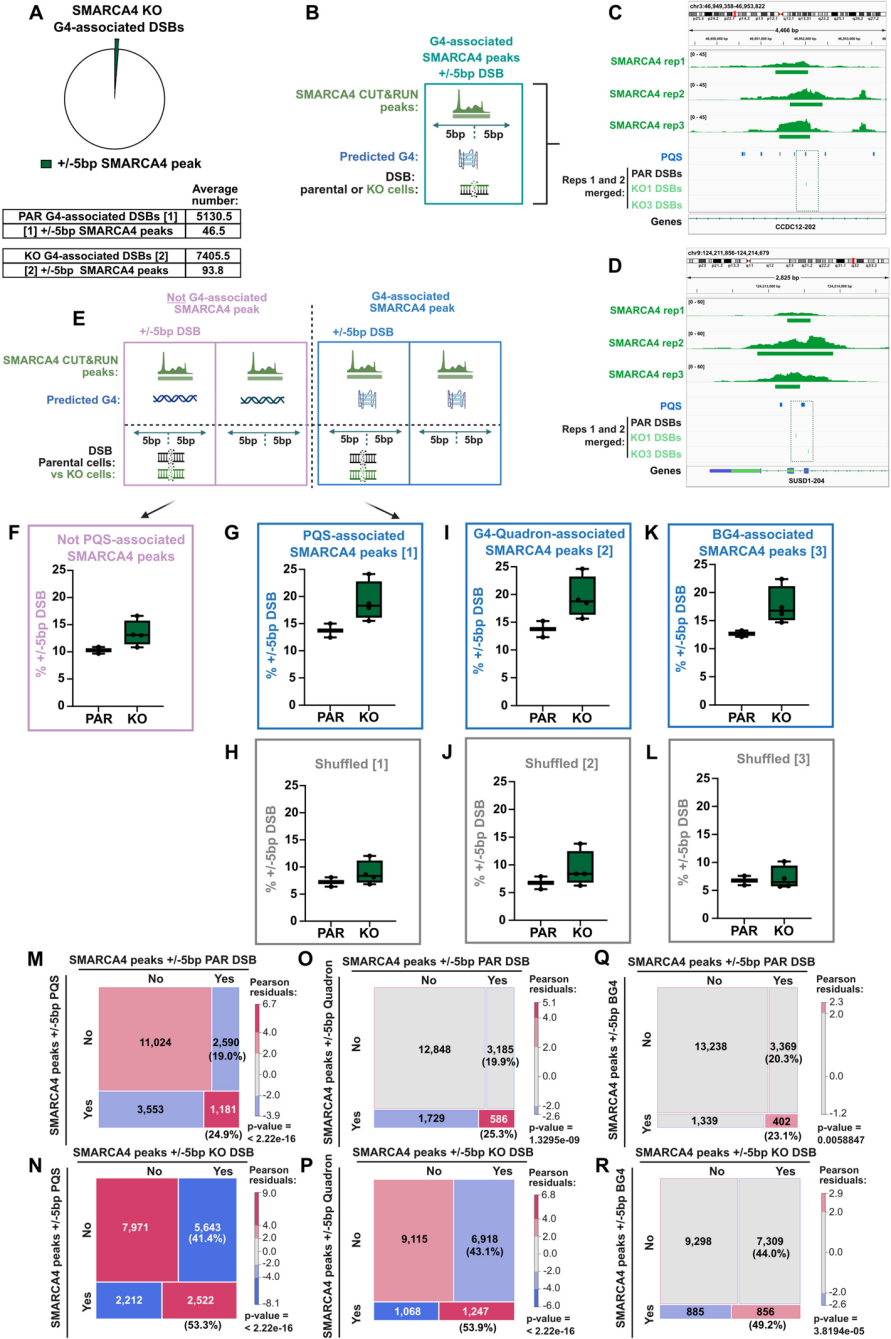
G4-associated SMARCA4 binding sites are more likely to have DSBs, in absence of SMARCA4. **A** Pie chart (top) indicating the proportion of G4-associated (± 5 bp PQS) *SMARCA4* KO DSBs that are within 5 bp of a SMARCA4 peak. A table (bottom) detailing the number of DSBs within a window of 5 bp of PQS (with average of parental (PAR) or KO clone replicates) and the number of those within 5 bp of a SMARCA4 peak. **B** Schematic representing G4-associated SMARCA4 peaks that are also within 5 bp of a DSB. **C-D** Representative genome tracks displaying read coverage from 3 independent biological replicates of SMARCA4 CUT&RUN (dark green). Predicted G4 sequences (PQS, blue) and PAR (black) and *SMARCA4* KO clone (light green) DSB coordinates are highlighted (two replicates of DSBs merged in each track). Gene annotations also shown. **E** Schematic representing SMARCA4 peaks that are Not G4-associated (left) or are G4-associated (right), subdivided by whether peaks are also within 5 bp of a DSB (bottom). **F-L** Box and whisker plots indicating percentage of SMARCA4 peak groups within a 5 bp window of DSBs, for parental DSBs (PAR) and *SMARCA4* KO clone DSBs (KO). SMARCA4 peak groups are defined as either Not PQS-associated (**F**), or those that are within a 5 bp window of predicted G4 sites (**G**: PQS-associated, **I**: G4-Quadron-associated, **K**: BG4-associated, or shuffled controls (**H**, **J**, **L)**. **M-R** Mosaic plots of observed frequencies of cooccurrence of SMARCA4 peaks within a 5 bp window of G4 sites (either PQS (**M**, **N**), G4 Quadron sites (**O**, **P**), or BG4 peaks (**Q**, **R**)), with either PAR DSBs (**M**, **O**, **Q**) or *SMARCA4* KO DSBs (**N**, **P**, **R**). Numbers of peaks in each subset are annotated, with percentages of peaks in each row that cooccur with DSBs. Tile sizes are proportional to the observed frequencies. Red indicates positive and blue indicates negative Pearson residuals from chi-square test. P-value indicates evidence for significant difference from expected values. Figures **B** and** E** were generated using BioRender

The fact that a substantial population of SMARCA4 binding occurs at G4s raises the possibility that the DSBs and genome instability that arises in the absence of SMARCA4 might be influenced by its normal association with these sites. To test this, we explored the relationship between G4-associated DSBs and SMARCA4 binding sites. Notably, we find that the vast majority of G4-associated DSBs identified in *SMARCA4* KO cells are in locations not normally bound by SMARCA4 (Fig. 4A and Additional file 1: Fig. S6A). This could be a consequence of transient or sporadic SMARCA4 association with these sites, such as in response to G4-induced replication stress (explored further below), or this could reflect an indirect effect of SMARCA4 on protecting G4s from damage.

Nevertheless, we interrogated the sites that are normally bound by SMARCA4 (Fig. 4B-E) to determine whether they are disproportionately prone to damage when SMARCA4 is deficient, and whether the presence of a G4 matters. When SMARCA4-bound sites that do not contain predicted G4s are analysed, we find that there are slightly more DSBs in the *SMARCA4* KO compared with the parental (Fig. 4E, F). However, this difference is greater when G4-proximal SMARCA4 sites are analysed with almost 20% of the SMARCA4-bound G4 sites having a DSB in the *SMARCA4* KO cells, which is greater than expected by chance (Fig. 4G-L).

Using Pearson chi-square analysis of these data, we find that the likelihood of a G4-associated DSB occurring at a site where SMARCA4 is normally bound is much greater in cells lacking SMARCA4 (Fig. 4M-R), suggesting that these sites are indeed protected by SMARCA4. Notably, this pattern is apparent when using any of the three G4 datasets (Fig. 4M-R).

When SMARCA4 peaks are analysed for their relationship with other non-B form DNA structures, we find that there is an enrichment of DSBs at these sites in the *SMARCA4* KO cells when compared with parental (Additional file 1: Fig. S6B-M). However, these trends are also apparent in the shuffled controls (Additional file 1: Fig. S6B-M). When the data are analysed using Pearson chi-square tests, many of these appear to be significant relationships in both the parental and *SMARCA4* KO cells (Additional file 1: Fig. S6N-Y). The structures that have altered patterns in these mosaic plots when SMARCA4 is deficient include short tandem repeats and direct repeats (Additional file 1: Fig. S6P, Q and Fig. S6V, W), suggesting that perhaps SMARCA4 also plays a protective role at these structures, but further work is needed to develop this possibility.

Analysis of the feature distribution associated with the subset of SMARCA4-bound G4-associated DSBs is like that of G4-associated DSBs, with a greater proportion in promoter regions (Additional file 1: Fig. S7A-F). Interestingly, the SMARCA4-bound DSB-containing sites not associated with G4s are much less clearly associated with promoter sequences, although the pattern is nevertheless still slightly different from the shuffled control (Additional file 1: Fig. S7E-H). As expected, the SMARCA4-bound DSB-containing G4-associated sites are more likely to be at promoters, and these sites are enriched for H3K4me3 and H3K9ac marks, although no difference in these patterns is apparent between the DSBs associated with sites normally bound by SMARCA4 in parental or *SMARCA4* KO cells (Additional file 1: Fig. S7I-L).

Taken together, while representing a minority of DSBs, the G4-containing sites where SMARCA4 is normally bound are disproportionately vulnerable to DSB formation when SMARCA4 is deficient, suggesting that its presence is protective.

### SMARCA4-deficient cells show sensitivity and changes in DNA damage responses when exposed to the G4 stabilising ligand pyridostatin

G4s can be stabilized by compounds that interact with these structures, such as pyridostatin (PDS; [37]). When treated with PDS, cells show increased levels of transcription- and replication-dependent DNA double strand breaks (DSBs) and growth arrest [16]. Cells with defects in DSB repair pathways are particularly sensitive to PDS treatment (for example, [17, 18]), and this has been developed in the clinic for targeting BRCA-deficient cancers [38, 39].

**Fig. 5:**
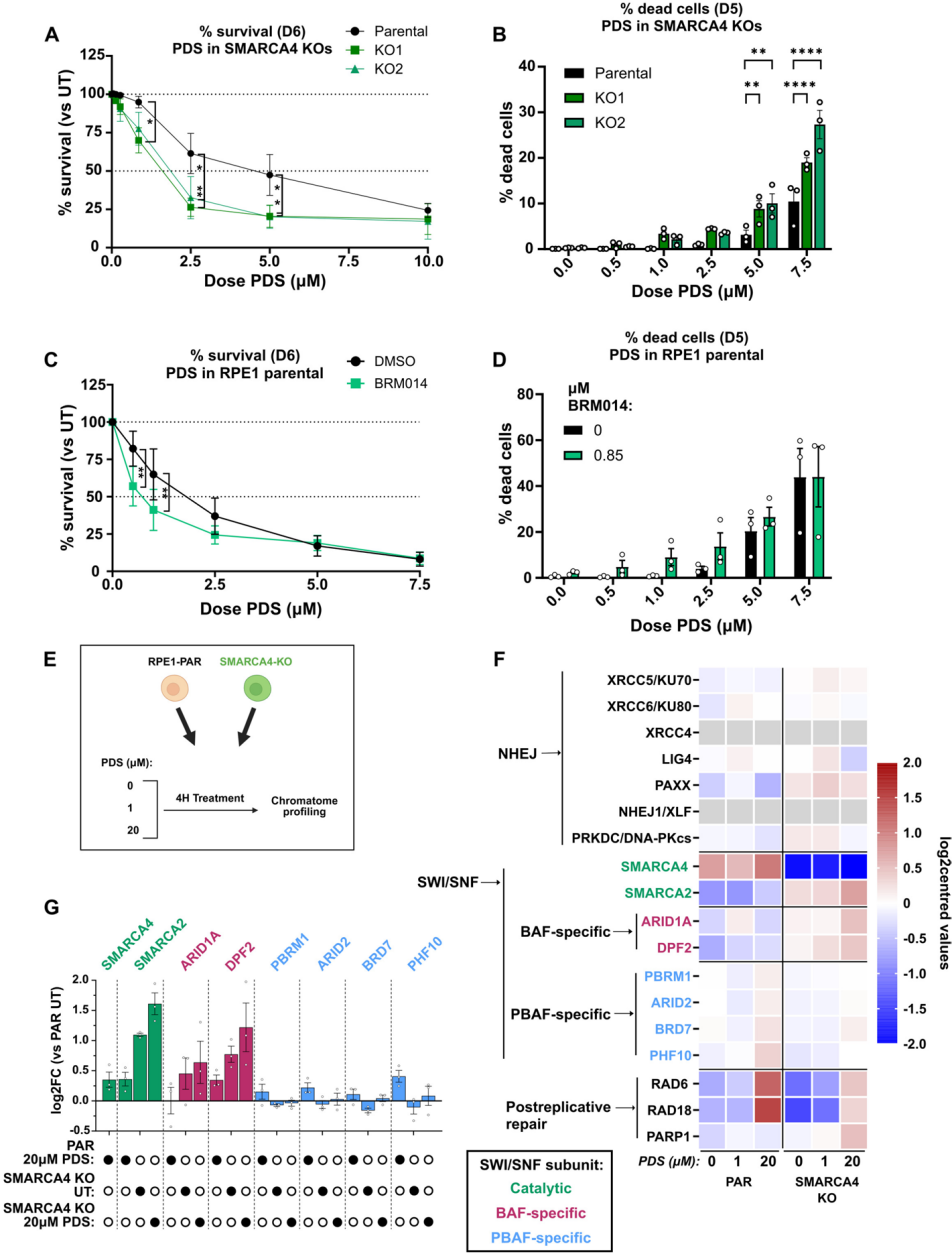
*SMARCA4* knockout cells are sensitive to the G4 stabilising ligand pyridostatin. **A** Percentage survival following treatment with increasing doses of PDS (μM) versus untreated cells, for 6 days (D6), in RPE1 parental and *SMARCA4* knockouts, *n* = 3. **B** Quantification of % of dead cells, defined as % of propidium-iodide positive cells in a cell population, after 5 days (D5) in culture, with increasing doses of PDS, in RPE1 parental and *SMARCA4* knockouts, *n* = 3, with points indicating independent biological replicates. For **A**,** B**, Data were analysed by two-way ANOVA with Tukey’s multiple comparisons test. **C** Percentage survival of parental RPE1 cells ± BRM014 (0.85 µM) with increasing doses of PDS, after 6 days in culture (D6), *n* = 4. **D** Quantification of % of dead cells, defined as % of propidium-iodide positive cells in a cell population, after 5 days in culture (D5), with increasing doses of PDS, in RPE1 parental cells with or without treatment with ATPase inhibitor BRM014 (0.85 µM), *n* = 3, with points indicating independent biological replicates. For **C**,** D**, data were analysed by two-way ANOVA with Šídák's multiple comparisons test. For **A-D**, mean ± SEM was plotted, *p < 0.05, **p < 0.005, ****p < 0.0001. **E** Schematic indicating setup for chromatome profiling. Figure was generated using BioRender. **F** Heatmap showing proteins on chromatin, grouped by function, represented as log2centred values, in untreated conditions or following treatment with PDS (1 or 20 μM, 4 h). **G** Mean ± SEM log2 fold changes (Log2FCs) of SWI/SNF subunit protein abundance in the chromatin-bound fraction are plotted for each condition versus the untreated control in parental RPE1 cells. The order of log2FC for each subunit is indicated with solid circles below, and as follows, for cells following 4 h treatment with 20 μM PDS in parental (PAR 20 μM PDS), or *SMARCA4* KO untreated (UT) or following 4 h treatment with 20 μM PDS (20 μM PDS). Points correspond to independent biological replicates

To see whether the absence of SMARCA4 impacted on cellular sensitivity to G4 binding ligands, we performed survival assays and found that the *SMARCA4* KO cells were more sensitive to treatment with PDS than the parental cells (Fig. 5A). Monitoring the presence of dead cells following PDS treatment similarly showed a vulnerability of *SMARCA4* KO cells (Fig. 5B). To determine whether the catalytic activity of SMARCA4 is required for survival after PDS exposure, we used the BRM014 inhibitor [40]. This inhibitor also targets the SMARCA4 paralogue SMARCA2, which is, in many cell lines, synthetic lethal with SMARCA4 (e.g. [41]). Consistent with this, we find that our *SMARCA4* KO lines (expressing only SMARCA2) are more sensitive to BRM014 than the SMARCA2 and SMARCA4 proficient parental cells (Additional file 1: Fig. S8A). We therefore tested survival of the parental cells to low doses of BRM014 and found a dose, 850 nM, that has minimal impact on survival in the parental (Additional file 1: Fig. S8A-C). When cells are grown in the presence of 850 nM BRM014, they show decreased survival and increased cell death following PDS exposure when compared to those grown without the inhibitor (DMSO control; Fig. 5C, D). These data are consistent with SMARCA4 preventing PDS-induced cell death through chromatin remodelling activity.

The cytotoxicity of PDS has been shown to be mediated by its ability to induce DNA damage [42, 43]. We therefore monitored phosphorylation of H2AX (to form gH2AX) as a readout of DNA DSBs and found that numbers of foci were significantly increased to similar levels in both the parental and the *SMARCA4* KO cells at early time points following PDS treatment and there was no difference in foci intensity (Additional file 1: Fig. S8D-F), suggesting that PDS does not produce a greater burden of DNA damage in the *SMARCA4* KO cells.

This raises the possibility that the *SMARCA4* KO cells are less able to respond to PDS-induced damage. PDS creates damage through both replication-dependent and -independent mechanisms [16]. The replication-independent mechanism is largely driven by PDS-dependent trapping of TOP2 molecules, and after processing, the resulting DSBs can be repaired by non-homologous end joining (NHEJ) [44]. Recently, using isogenic HCT116 and TOV21G cell line models, we demonstrated that there is a striking increase in chromatin-associated NHEJ proteins following treatment with PDS [45]. Moreover, we found that ARID1A-deficient cells are unable to mobilise NHEJ proteins to similar levels on chromatin and that this impairs their ability to survive [45], raising the possibility that the PDS sensitivity in the SMARCA4-deficient cells similarly stems from a defect in NHEJ mobilisation. We therefore performed chromatin profiling of the parental and *SMARCA4* KO cells after 4 h treatment with either 1 or 20 mM PDS (Fig. 5E). Surprisingly, we found that NHEJ proteins are not substantially increased in the chromatin fraction in either the parental or *SMARCA4* KO cells (Fig. 5F). While NHEJ is known to play a role in RPE1 responses to PDS [42], these data suggest that at least at these doses and time points, NHEJ is not mobilised onto chromatin to the same extent as in the cancer-derived TOV21G and HCT116 cell line models.

However, in support of a role for SWI/SNF in responding to PDS treatment, the chromatin association of SMARCA4 is increased at the higher dose of PDS. Interestingly, increased SMARCA2 chromatin enrichment is apparent in the untreated SMARCA4 KO cells, and this shows further enrichment after PDS treatment (Fig. 5F, G), suggesting that perhaps it partly compensates for SMARCA4 loss in response to PDS. Moreover, ARID1A and another BAF-specific subunit DPF2 are more enriched in the chromatin of PDS-treated *SMARCA4* KO cells when compared with parental cells, whereas the PBAF-specific subunits are very slightly enriched in the chromatin of PDS-treated parental but not PDS-treated *SMARCA4* KO cells (Fig. 5F, G) Notably, in the absence of any treatment, there is a slight increase of SMARCA2, along with ARID1A and DPF2 on chromatin in the *SMARCA4* KO cells (Fig. 5F, G), which could indicate compensatory activity when SMARCA4 is deficient. Together, these data raise the possibility that SMARCA4-containing PBAF complexes respond to PDS in RPE1 cells, and when SMARCA4 is deficient, SMARCA2-containing BAF complexes, which may already be compensating for SMARCA4 loss even before treatment, respond instead.

To understand whether SMARCA4 loss changes the response to PDS-induced DNA damage beyond NHEJ, we looked at proteins involved in DNA repair and replication and noticed that the post-replicative repair proteins RAD6 and RAD18 fail to associate to the same levels with chromatin in response to PDS in the *SMARCA4* KO cells (Fig. 5F). This could suggest a defect in replication-associated damage responses. If correct, one prediction would be increased single stranded DNA, and in support of this, we find more PARP1 in the chromatin of PDS-treated SMARCA4 KO cells (Fig. 5F).

**Fig. 6:**
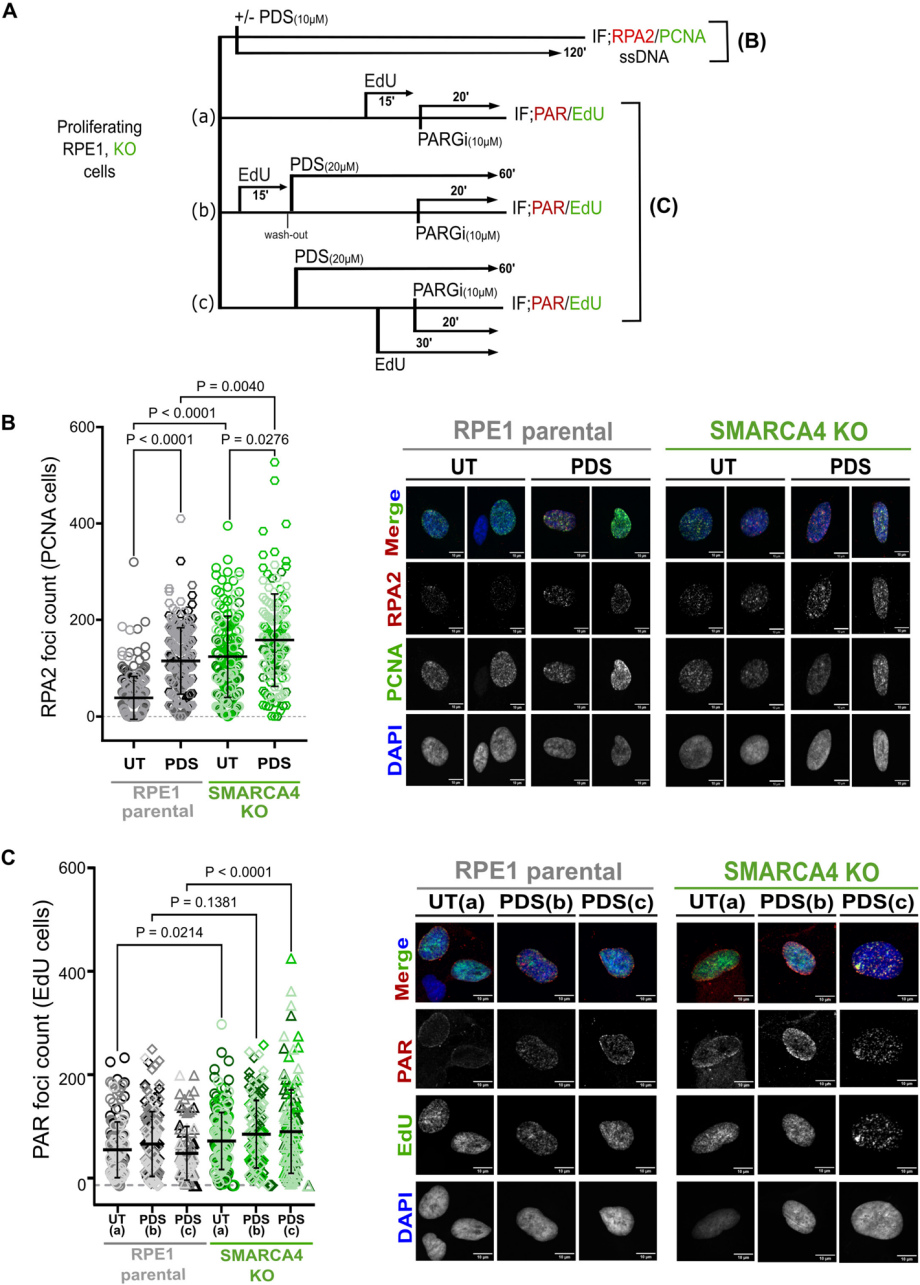
*SMARCA4* knockout cells have accumulation of ssDNA gaps in S-phase. **A** Schematic showing the ssDNA detection assay workflow, including PDS treatment, EdU pulsing, and PARG inhibitor (PARGi) addition timing. RPA2 detection in combination with PCNA identifies ssDNA in S-phase cells under unperturbed or PDS-treated conditions. PARGi was added 20 min before fixation to stabilize transient PAR whereas PCNA and EdU are markers of S-phase cells. Different timings of EdU pulses for detecting PAR in untreated cells and in response to PDS are labelled as (a), (b) or (c).** B** Quantification of RPA2 foci per PCNA-positive cells in RPE1 parental or *SMARCA4* knockout cells under unperturbed or PDS treatment (10 µM, 2 h). **C** Quantification of PAR (poly[ADP-ribose]) foci in EdU-positive cells in RPE1 parental or SMARCA4 knockout cells under unperturbed or PDS treatment (20 µM, 1 h). For **B** and **C**, independent biological replicates are represented by different shades of grey for parental RPE1, or green for *SMARCA4* KO cells (*n* = 4/3). EdU pulse timings, as described in **A**, are indicated in brackets. Error bars indicate mean ± SD, and statistical significance was assessed using a Kruskal–Wallis test with multiple comparisons. Only significant p-values are plotted. Representative images of RPA2/PCNA and PAR/EdU immunofluorescence are shown on the right. Scale bar represents 10 µm

To look at this more directly, we monitored single stranded DNA using RPA foci as a readout. Cells were treated with PDS, and immunofluorescence was performed with antibodies against RPA (Fig. 6A). PCNA was analysed in parallel to identify cells in S phase. We found that the number of S phase RPA foci is higher in the absence of SMARCA4 even under untreated conditions (Fig. 6B), suggesting that they might already have increased stalled forks or single strand gap formation during replication. RPA foci numbers increase in both the parental cells and the *SMARCA4* KO cells in response to PDS, with the largest number in the *SMARCA4* KO cells (Fig. 6B).

The increased PARP1 in the chromatin of PDS-treated *SMARCA4* KO cells (Fig. 5F) is consistent with the possibility that there are more stalled forks or single stranded gaps in these cells compared with parental. To shed light on this, we analysed the numbers of poly-ADP ribose (PAR) foci in PDS-treated parental and *SMARCA4* KO cells. The cells were stained with EdU to identify replicating cells, using a pulse of EdU either prior to or during PDS treatment to ensure that we captured both ongoing replication and DNA synthesis at stalled forks or persistent single-stranded gaps (Fig. 6A). We found that the numbers of PAR foci were increased in the *SMARCA4* KO cells relative to the parental, both in the untreated cells and following PDS treatment (Fig. 6C). Together, these data are consistent with a role for SMARCA4 during replication that prevents single stranded DNA formation and mutagenesis, particularly at sites of G4s.

### SMARCA4-deficient cancer samples are more likely to have genome instability at G4 sequences when compared with SMARCA4-proficient cancer samples

We performed our studies in RPE1, which is a non-cancer derived cell line. SMARCA4 is frequently misregulated in cancers, but it is unclear whether the relationship with genome stability at G4 sequences plays a role in SMARCA4-deficient cancers. To investigate this, we downloaded sequencing data from cancer patients [46, 47] and identified samples with mutations in *SMARCA4*. We further separated out samples with mutations in *TP53*, since this is known to have an impact on genome instability, including at G4 sequences [20]. Samples with both *SMARCA4* and *TP53* mutations were excluded to avoid confounding effects. The remaining samples were those lacking mutations in either *SMARCA4* or *TP53* (‘Not’).

**Fig. 7:**
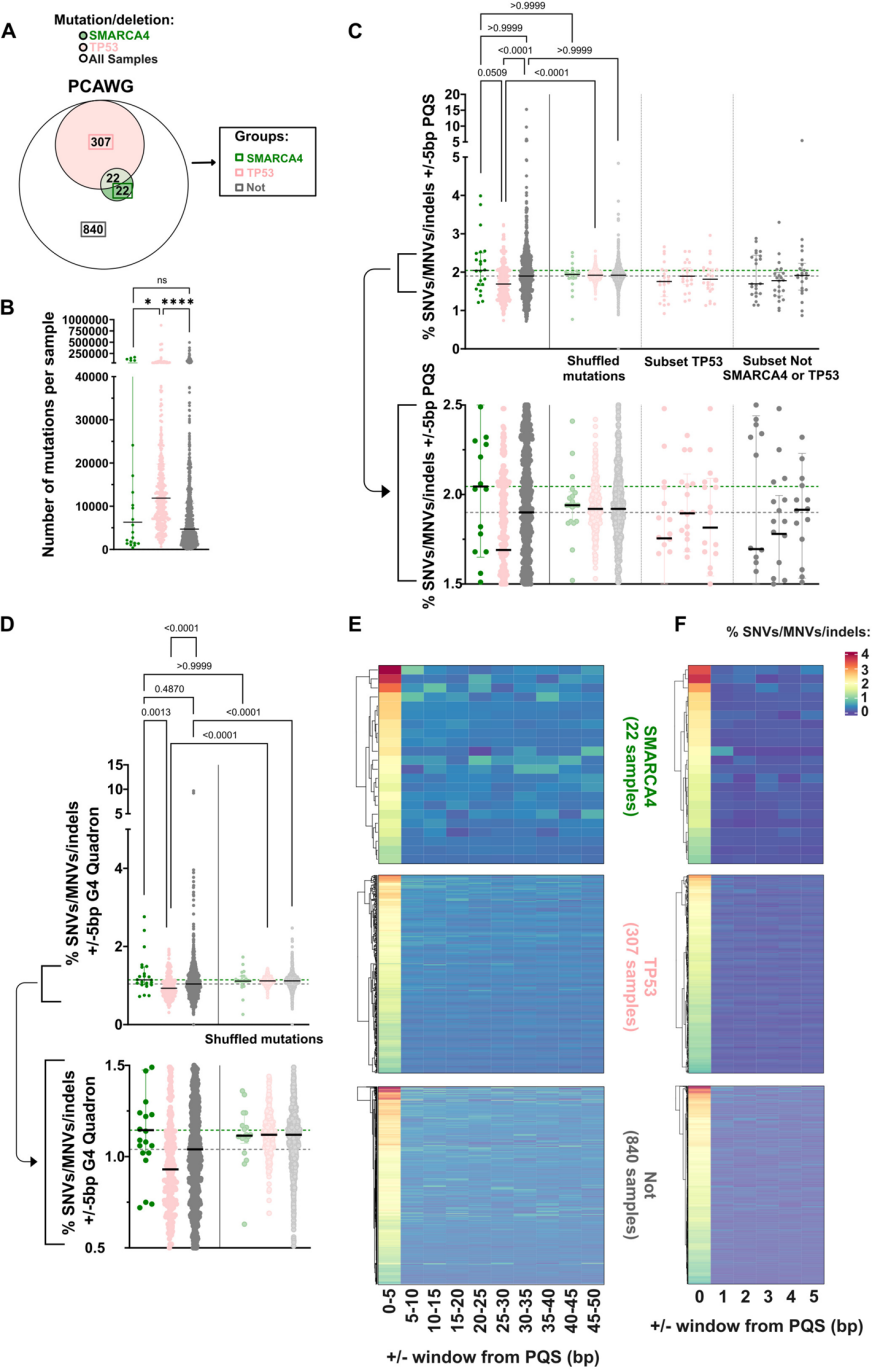
Cancer samples with *SMARCA4* mutation/deletion are more likely to have mutations near predicted G4s. **A** Venn diagram indicating the overlap of PCAWG patient samples (white) with either mutation or deletion of *SMARCA4* (green) or *TP53* (pink). Patient sample groups are defined as in the box, with boxes around the numbers in the Venn diagram that correspond to the number of samples in each group. **B** The number of mutations per sample in PCAWG patients, separated by mutation status, with each sample represented as an individual point, and a line at the median. **C**,** D** Scatter plots indicating percentages of SNVs/MNVs/indels within a 5 bp window of PQS sites (**C**) or G4-Quadron sites (**D**) in PCAWG samples, grouped by mutation status (as defined in **A**), with each point representing an individual patient sample and a line for the median (top panel), with a zoomed inset (bottom panel). Dotted lines are plotted for the median percentage of the *SMARCA4* mutant samples (green), and the samples with Not *SMARCA4* or *TP53* mutation/deletion (grey). **E**,** F** Hierarchical heatmaps representing the percentage of SNVs/MNVs/indels that are within a window of PQS sites, either shown in 5 bp bins from 0–50 bp (**E**) or in 1 bp bins from 0–5 bp (**F**) for PCAWG patient samples labelled and plotted separately for each patient group. For **B-D**, statistical significance was assessed using a Kruskal–Wallis test with Dunn’s multiple comparisons test (for **B**, *: < 0.05 and ****: < 0.0001)

Using data from a pan-cancer whole genome sequencing study [46], we found that *SMARCA4*-mutant samples had a lower median number of mutations (SNVs, MNVs or indels) per sample when compared with the *TP53*-mutant group, at a level that was comparable to the group without mutations in either gene (Fig. 7A, B). When these were intersected with predicted G4 sites using PQS or Quadron, we found that the percentage of G4-associated mutations is highest in the *SMARCA4* mutant group (Fig. 7C, D), and these were significantly higher when compared with the *TP53*-mutant group when analysed with Quadron G4s (Fig. 7D). The differences between the three groups were minimal when the intersection was performed using shuffled controls (Fig. 7C; ‘shuffled mutations’). Because the sample numbers in the *SMARCA4*-mutant group were small, we also used a subsampling approach and randomly selected the same number of samples from the two larger groups and performed the same analysis. Notably, the subsampled groups still showed a lower percentage of G4-associated mutations than that found with the *SMARCA4*-mutant population (Fig. 7C; ‘subset TP53’ and ‘subset Not SMARCA4 or TP53’).

Again, we tested non-B form DNA structures, including A phased repeats, direct repeats, inverted repeats, mirror repeats, short tandem repeats, and Z-DNA motifs (Additional file 1: Fig. S9A-F). For these structures, we found that there were no significant differences in the overlap between mutations and structures in the *SMARCA4*-mutant group when compared with the other two groups, suggesting that SMARCA4 specifically protects genome stability at G4-forming sequences.

We also used sequencing datasets from TCGA from melanoma (skin cutaneous melanoma, SKCM) [47]. The samples were filtered by mutational status as before (Additional file 1: Fig. S9G). Similar to the pan-cancer analysis, we found that *SMARCA4*-mutant samples had a lower median number of mutations per sample when compared with *TP53*-mutant samples (Additional file 1: Fig. S9H). The *SMARCA4*-mutant samples showed more G4-associated mutations than would be expected by chance using PQS, but this was also true for the other two groups in the SKCM samples (Additional file 1: Fig. S9I). Using Quadron-predicted G4s instead, all three groups still showed an increased percentage of G4-associated mutations over shuffled controls, but in this case, the *SMARCA4*-mutant group was noticeably higher than the other two groups (Additional file 1: Fig. S9J).

We next analysed the proximity of the mutations relative to G4s using either PQS or Quadron datasets. To do this, we divided the mutation data into either 5 or 1 bp windows moving away from the G4. In all patient groups, the frequency of mutations is greatest nearest the G4 and drops off within 1 bp (Fig. 7E, F and Additional file 1: Fig. S10A, B), again suggesting that the mutations directly intersect with G4-forming sequences.

The patterns of mutations in patient data are consistent with the cell line model-derived analyses and suggest that G4s are vulnerable to increased genome stability. Moreover, SMARCA4 plays a protective role, and patients with SMARCA4-deficiency, at least in some contexts, have a modestly higher level of G4-associated mutations.

## Discussion

**Fig. 8:**
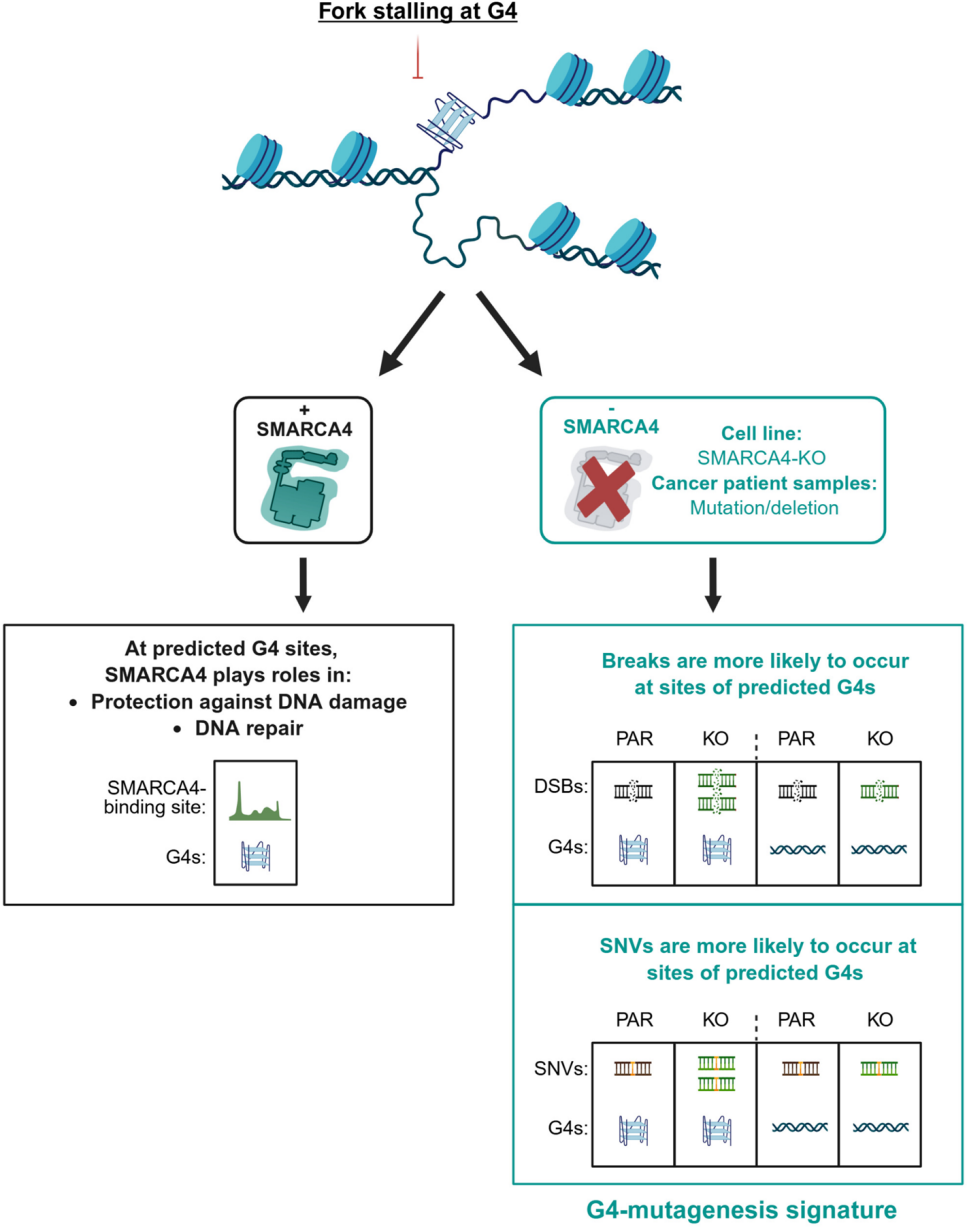
A G4-mutagenesis signature is found in SMARCA4-deficient samples. A schematic detailing the role of SMARCA4 at predicted G4-sites, and the effects on genome stability at these sites when SMARCA4 is lost or the gene is mutated, either in cell line models or cancer patient samples

We find that SMARCA4 plays a protective role and prevents DNA breaks and mutations at G4 sequences in the human genome. This is apparent both in cell line models and through an analysis of whole genome sequencing data from cancer samples (Fig. 8). This protective activity could play a role during cancer progression. *SMARCA4* loss-of-function mutations are frequent and can occur early during tumourigenesis, which means that the evolution of cancer following SMARCA4 loss is likely to result in increased instability around G4 forming sequences.

Notably, most of the differences uncovered in this study are relatively modest. However, because of the relationship between G4s and regulation of gene expression, the consequence of modest changes at G4 sequences could be functionally very important. Changes to G4 sequences that regulate oncogenes or tumour suppressor genes could lead to their transcriptional misregulation, which could impact on tumourigenesis or cancer progression. Of note, a single base change can alter the ability of a sequence to form a G4 [22, 48]. In addition, defects in replicating regions of the genome that contain G4-forming sequences can lead to epigenetic instability [49, 50]. An interesting possibility, therefore, is that SMARCA4 absence impacts not only genome stability, but epigenetic stability, in these regions.

A major outstanding question is how SMARCA4 functions to protect the integrity of G4-containing sequences. There are multiple activities that could potentially play a role, and these are not mutually exclusive. For example, SMARCA4-dependent remodelling at chromatin containing G4s could promote their resolution and facilitate progression of polymerases. This could occur through creating accessibility for G4-resolving helicases, or through regulating the assembly or disassembly of RNA loops, which have recently been shown to regulate G4 structures [51]. This latter possibility is compelling given the known relationship between SMARCA4 and transcriptional regulation at DNA double strand breaks and R loop homeostasis [28, 52]. Alternatively, SMARCA4 might play a role in the processing or resolution of G4-stalled replication forks or protecting vulnerable structures during remodelling.

Importantly, the absence of SMARCA4 creates a vulnerability to treatment with G4 stabilising ligands. Since these are now being used in the clinic [38, 39, 53], this presents a potential new therapeutic approach for patients with SMARCA4-deficient tumours.

## Conclusions

G4 structures are vulnerable to genetic changes, and here, we show that the SMARCA4 subunit of the SWI/SNF chromatin remodelling complex is a key protective factor. Using sequencing-based approaches in isogenic cell line models, we demonstrate that cells lacking SMARCA4 have more DNA double strand breaks at G4-forming sequences. This difference is even more striking when examining SMARCA4-bound G4 sites, suggesting that its direct association in chromatin plays a protective role. Moreover, the level of G4-associated alterations is higher, both in isogenic cell line models and patient samples, when SMARCA4 is deficient. Cell biology assays show an altered molecular response and decreased survival after treatment with the G4-binding ligand pyridostatin when SMARCA4 is absent. These data highlight the vulnerability of G4 structures and the importance of SMARCA4 in their protection, particularly in the context of cancer.

## Methods

### Cell culture

hTERT-RPE1 (RPE1, source: ATCC, not authenticated further) were cultured in Dulbecco modified minimal essential medium (DMEM)/F-12 (Merck) supplemented with 10% FBS (Gibco), 200 µM glutamax (Gibco), 0.26% sodium bicarbonate (Gibco), and 1% penicillin/streptomycin (P/S; Merck). Cells were maintained at 37 °C in a humified incubator with 5% CO2 and were regularly tested for mycoplasma contamination.

### Generation of knockouts using CRISPR/Cas9-mediated gene editing

*SMARCA4* knockouts generated in RPE1 cells (source: ATCC, not further authenticated) were described previously [54]. Resulting clones were screened for loss of protein expression using Western blotting, followed by Sanger sequencing of the targeted genomic region, as well as immunofluorescence, microscopy and proteomic profiling.

### INDUCE-seq library construction

The detailed library construction protocol and data processing pipeline have been published [35]. Briefly, 1X10^5 cells were seeded to each well of a 96-well plate pre-coated with Poly-L-lysine and fixed in 4% PFA for 10 min at room temperature. Cells were subjected to the two-step permeabilisation as described in the protocol and washed three times in 1xCutSmart® Buffer (NEB). The DNA double strand ends were end repaired with Quick Blunting Kit (NEB) and A-tailed with NEBNext® dA Tailing Module (NEB) according to manufacturer’s protocol. A-tailed ends were ligated to the modified P5 adapter and excessive P5 adapter was washed away after ligation. Genomic DNA was extracted with Genomic DNA Clean & Concentrator™ (Zymo), sonicated to 300–500 bp fragments, and size selected to exclude fragments smaller than 150 bp.Fragmented and size-selected DNA was then end-repaired and ligated to staggered P7 adapter with NEB Next® Ultra™ II Ligation Module (NEB) according to the manufacturer’s protocol. The ligated sequencing libraries were size selected to remove fragments smaller than 200 bp and residual P7 adapter. Samples were pooled and concentrated before sequencing on an Illumina NextSeq 500 platform with 75 bp single-ended reads.

### INDUCE-seq data processing

For the data processing pipeline, FASTQ files were demultiplexed and adapter sequences were removed with Trim Galore (v0.6.6) (Krueger F, Trimgalore (2023), GitHub repository, https://github.com/FelixKrueger/TrimGalore) using the options -f fastq -e 0.1 -q 20 -O 1 -a AGATCGGAAGAGC. Reads were mapped to human reference genome (CHM13-T2Tv1.1, GenBank GCA_009914755.3) using BWA-MEM (v0.7.17, Li H. (2013) Aligning sequence reads, clone sequences and assembly contigs with BWA-MEM. arXiv:1303.3997v2 [q-bio.GN). Mapped reads were quality filtered with SAMtools (v1.11) [55] and a custom script [35] and then converted into BED files. Centromere sequences (except the ct arms) and ChrM were excluded for further analysis. The following awk script was used to exclude ct arms from the censat annotation file for filtering: awk '$4! ~/^ct/'. The breakend position was recorded as the first 5’ nucleotide relative to the strand orientation and optical read duplicates were removed with a custom script [35].

### Whole genome sequencing

RPE1 parental and *SMARCA4* KO clones KO1 and KO3 were grown for 1 month, followed by single cell sorting into 96-well plates using a FACSAria™ III sorter (BD). Three clones of RPE1 parental and six clones of each *SMARCA4* KO, as well as original pools of all three were used for whole genome sequencing. Genomic DNA was extracted using phenol/chloroform and precipitated in ethanol, then quantified using Qubit. 1 µg of DNA per sample was used as input material for sequencing library preparation. Libraries were created using the NEBNext DNA Library Prep kit following manufacturer's recommendations. Genomic DNA was fragmented to 350 bp by shearing. DNA fragments were end polished, A-tailed, and ligated with the NEBNext adapter for Illumina sequencing. Fragments were amplified by PCR with the P5 and indexed P7 oligos. Amplified libraries were purified with the AMPure XP system, then analysed for size distribution using the Agilent 2100 bioanalyzer and quantified using real-time PCR. Libraries were sequenced on the Illumina Novaseq 6000 with 150 × 150 bp reads.

### Whole genome sequencing analysis

Fastq reads were trimmed using Trimmomatic using the options LEADING:25 TRAILING:25 MINLEN:50 SLIDINGWINDOW:10:25 ILLUMINACLIPTruSeq3-PE.fa:2:30:10 – then aligned with BWA-MEM (v0.7.17) to CHM13-T2Tv2.0 (https://console.cloud.google.com/storage/browser/gcp-public-data--broad-references/t2t/v2/chm13v2.0.maskedY.rCRS.EBV.fasta). Sam files were cleaned and converted to bam format using Picard tools (v2.23.8) (“Picard Toolkit.” 2019. Broad Institute, GitHub Repository. https://broadinstitute.github.io/picard/; Broad Institute) Cleansam function. Bam files were sorted using Samtools (v1.11) sort, and then processed using Picard tools (v2.23.8) AddOrReplaceReadGroups and MarkDuplicates functions, followed by GATK (v4.1.9.0, [56]) BaseRecalibrator and ApplyBQSR functions. GATK (v4.1.9.0) mutect2 was used to call somatic mutations of WGS from clones cultured for a month versus the original stock (e.g. KO1 clone 1 vs KO1_parental, or WT clones 3 vs WT_parental), using a SNP file downloaded from UCSC (https://hgdownload.soe.ucsc.edu/gbdb/hs1/dbSNP155/chm13v2.0_dbSNPv155.vcf.gz) as the panel of normals. These somatic mutations were filtered and annotated using GATK (v4.1.9.0) FilterMutectCalls and SelectVariants, SnpSift (v4.3t, [57]), BCFtools (v1.11, [55]) isec and norm functions, and SnpEff (v4.3t, [58]). The bedops (v2.4.39 [59],) vcf2bed function was used to extract SNV, insertion and deletion coordinates from the vcf files to separate bed files for downstream analysis.

### ATAC-seq experimental protocol

ATAC-seq (assay for transposase-accessible chromatin by sequencing) was performed according to the Omni-ATAC protocol [60]. Briefly, 1 × 10^5^ cells were collected per experiment, and nuclei were extracted. Nuclei were incubated with Tn5 on ice for 30 min. DNA was purified using the Qiagen MinElute reaction cleanup kit (Qiagen) and amplified using the NEBNext high-fidelity PCR master mix (New England Biolabs) and Illumina Nextera DNA indexes (Illumina). Libraries were purified using AMPure XP beads (Beckman Coulter), then profiled using the Agilent TapeStation D1000 high sensitivity ScreenTape on the Agilent 4150 TapeStation System and sequenced on the Illumina Novaseq 6000 with 150 × 150 bp reads by Novogene (Novogene Corporation Inc. UK).

### ATAC-seq analysis

Fastq reads were trimmed using TrimGalore (v0.6.6) using the options –trim-n –paired. Trimmed reads were mapped to the human CHM13-T2Tv1.1 genome (GenBank GCA_009914755.3) using Bowtie2 (v2.4.2) [61] using the parameters –local –very-sensitive-local –no-unal –no-mixed –no-discordant –dovetail –soft-clipped-unmapped-tlen –non-deterministic –phred33 -I 50 -X 1000. Reads with more than 3 mismatches were removed with Sambamba (v0.5.0) [62], and their corresponding mates were removed with Picard tools (v2.23.8). Sam files were converted to bam with SAMtools (v1.11). Reads mapped to chrM were removed using SAMtools, and duplicate reads were removed using Picard tools (v2.23.8). Bigwig files were generated using deepTools bamCoverage [63]. Significant peaks were called using MACS2 (v2.2.7.1) [64] callpeak with filtered bam files as input and options -g 3,054,832,041 -f BAMPE −0.01. Bedtools (v 2.29.2) [65] multiinter and merge functions were used to merge peaks that were called in both reps.

### SMARCA4 CUT&RUN data processing

RPE1 parental SMARCA4 (GSM7498446-7,498,451), IgG (GSM7498464-7,498,469) and SMARCA4-KO SMARCA4 (GSM7498490, GSM7498491) CUT&RUN datasets were obtained from GEO (GSE235294) [54]. Fastq reads were trimmed using TrimGalore (v0.6.6) using the options –trim-n –paired. The human CHM13-T2Tv1.1 (GenBank GCA_009914755.3) and S. cerevisiae S288C (GenBank GCA_000146045.2) assemblies were combined into one FASTA file, then trimmed reads were mapped using Bowtie2 (v2.4.2) using the parameters –local –very-sensitive-local –no-unal –no-mixed –no-discordant –dovetail –soft-clipped-unmapped-tlen –non-deterministic –phred33 -I 50 -X 1500. Reads with more than 3 mismatches were removed with Sambamba (v0.5.0), and their corresponding mates were removed with Picard tools (v2.23.8). Sam files were converted to bam with SAMtools (v1.11). Reads mapped to the CHM13-T2Tv1.1 assembly were extracted using BAMtools (v2.5.1) [66] split. Duplicate reads were removed using Picard if deemed necessary. Low and high salt BAM files from each sample were merged, sorted, and indexed using SAMtools. Significant peak calling was performed on uniquely mapping read bam files using MACS2 (v2.2.7.1) [64] callpeak using IgG as control, with options -g 3,054,832,041 -f BAMPE –keep-dup all -q 0.01 –broad –broad-cutoff 0.01. Peaks within centromere sequences (https://s3-us-west-2.amazonaws.com/human-pangenomics/index.html?prefix=T2T/CHM13/assemblies/annotation/chm13v2.0_censat_v2.0.bed) except the ct arms (using the following awk script to exclude ct arms from the censat annotation file: awk '$4! ~/^ct/'), and ChrM were excluded for further analysis, using bedtools. Bedtools (v 2.29.2) multiinter and merge functions were used to merge peaks that were called in at least 2 reps.

### BG4 CUT&TAG analysis

HEK293TN BG4 (GSM5395699) and IgG (GSM5395700) CUT&TAG datasets were obtained from GEO (GSE178668) [32]. Fastq reads were trimmed using TrimGalore (v0.6.6) using the options –trim-n –paired. Trimmed reads were mapped The human CHM13-T2Tv1.1 reference genome assembly (GenBank GCA_009914755.3) using Bowtie2 (v2.4.2) using the parameters –local –very-sensitive-local –no-unal –no-mixed –no-discordant –dovetail –soft-clipped-unmapped-tlen –non-deterministic –phred33 -I 10 -X 700. Reads with more than 3 mismatches were removed with Sambamba (v0.5.0), and their corresponding mates were removed with Picard tools (v2.23.8). Sam files were converted to bam then indexed with SAMtools (v1.11). Bigwig files were generated from bam files using deepTools bamCoverage –extendReads –binSize 1 and scaled according to the total number of mapped reads. Significant BG4 peak calling was performed using MACS2 (v2.2.7.1) callpeak using the BG4 bam file and IgG as negative control, with options -g 3,054,832,041 -f BAMPE –keep-dup all -q 0.00001 –broad –broad-cutoff 0.00001.

### Histone PTM ChIP-seq analysis

Histone mark ChIP-seq data for RPE1 cells across the cell cycle, and associated input data was downloaded from GEO accession GSE175752 [67]. Fastq files across the cell cycle (G1, ES, LS, G2) in RPE1. Ctrl cells were merged for downstream analysis. This dataset was analysed as above for CUT&RUN with a few exceptions, as they have single-end reads rather than paired-end reads, the Picard tools step to remove corresponding mates following removing reads with 3 mismatches was no longer required. Bigwig files were generated with updated bamCoverage parameters to not include –maxFragmentLength, but to update to –extendReads 250. As above, significant peak calling was performed on uniquely mapping read bam files using MACS2 (v2.2.7.1) [64] call peak using IgG as control, with options -g 3,054,832,041 -f BAMPE –keep-dup all -q 0.01 –broad –broad-cutoff 0.01.

### Predicted G4 intersection analysis

Predicted G4 (PQS) sites were downloaded as bed files from https://pqsfinder.fi.muni.cz/genomes for hg19 and hg38, or manually identified for the CHM13-T2T genome, using PQSfinder (v2.24.0) [67] with default settings, for each chromosome individually. For CHM13-T2T, Rtracklayer (1.62.0) was used to convert coordinates to GRanges format and create a GRanges list, which was then exported out in gff3 format. Bedops (v2.4.39) was then used to convert the gff3 file to bed file format for subsequent analysis. Non-B DNA motifs for CHM13-T2T, including G4-Quadron were downloaded as bigBed files from https://s3-us-west-2.amazonaws.com/human-pangenomics/index.html?prefix=T2T/CHM13/assemblies/annotation/nonB/ and converted to bed files using UCSC package bigBedToBed (v377). UCSC liftOver package [68] was used to liftover Non-B DNA coordinates from CHM13-T2T to hg19 and hg38, using chain files (hs1ToHg19.over.chain.gz and hs1ToHg38.over.chain.gz, respectively). EPDnew promoter annotations were downloaded for hg38 [69], and SEdb2.0 typical and super enhancer annotations were downloaded for hTERT-RPE1 in the hg38 genome (http://www.licpathway.net/sedb). Super enhancer and typical enhancer files were merged for intersection analysis. UCSC liftOver package was used to liftover promoter and enhancer coordinates from hg38 to CHM13-T2T using the hg38-chm13v2.over.chain.gz chain file.

Bedtools (v2.29.2) window was used, with options -u -w 5, to find coordinates within 5 bp of predicted G4 sites, predicted non-B DNA sites or shuffled controls. Intersecting coordinates from this analysis were then used to also find intersections within a 5 bp window with other annotation files, including SMARCA4 peaks, enhancers, open chromatin (defined by ATAC-seq peaks) and histone PTM ChIP-seq peaks. Bedtools (v2.29.2) shuffle was used to shuffle these coordinates to create shuffled controls, excluding centromere sequence (except ct_arms, using annotation file described above). A custom awk script was used to count the occurrence of total coordinates, the occurrence of coordinates within a 5 bp window of a predicted G4 site, for example, and then the percentage of total coordinates within 5 bp of a predicted G4 site. Percentages, or calculated fold changes were then plotted in GraphPad Prism (v10.6.1) either as box and whisker plots, dot plots, heatmaps, stacked colour bar charts or pie charts. To calculate the percentage of DSBs or SNVs that were within different bins of distance from a predicted G4, bedtools (v2.29.2) window was used as above, but setting different window (-w) options as the maximum distance for each bin (e.g. -w 30 for 25–30 bp bin), and subtracting the number overlapping in the subsequent distance (e.g. -w 25 for 25–30 bp bin), before calculating percentages in each bin.. The ComplexHeatmap R package (v2.24.1) [70] was used to plot heatmaps with hierarchical clustering. For analysis of either consensus SMARCA4 peaks or for INDUCE-seq, where there are only two replicates in the parental control, contingency tables were created, with either each SMARCA4 peak as a separate event, or each DSB as a separate event (replicates combined, and KO clones combined). These contingency tables were plotted as mosaic plots using the vcd R package (v1.4–13). CHM13-T2T versions were used for analysis with RPE1 datasets (including INDUCE-seq, WGS and CUT&RUN data). For the cBioportal datasets, hg19 versions were used for analysing PCAWG and hg38 for TCGA SKCM.

Screenshots of bigwig files and peaks were taken from IGV (v2.11.9). The ChIPseeker R package (v1.40.0) [71] was used to analyse the genomic distribution of peaks across features. The intervene package (v0.6.5) [72] was used to calculate overlapping peak intersections and resulting Venn diagrams or upset plots were re-drawn using the ggVennDiagram R package (v1.5.2) [73].

### Download and analysis of cBioportal whole-genome- (WGS) or whole-exome-sequencing (WXS) datasets

The cBioportal [74] website was used to filter samples by whether they contained a mutation or deletion in either SMARCA4 or TP53, and to create groups based on this for *SMARCA4* mutant, *TP53* mutant or Not *SMARCA4* or *TP53* mutant samples from each of the following datasets, PCAWG WGS [46]—https://xenabrowser.net/datapages/?dataset=October_2016_all_patients_2778.snv_mnv_indel.maf.xena.nonUS&host=https%3A%2F%2Fpcawg.xenahubs.net&removeHub=https%3A%2F%2Fxena.treehouse.gi.ucsc.edu%3A443) or TCGA SKCM WXS (https://gdc-hub.s3.us-east-1.amazonaws.com/download/TCGA-SKCM.somaticmutation_wxs.tsv.gz) [47]. Clinical data was downloaded from cBioportal separately for each patient sample group and then the sample IDs from these groups were extracted using an awk script. The PCAWG MAF file (containing filtered SNVs, MNVs and indels that had been aligned to the hg19 genome) and the TCGA tsv file (containing filtered somatic mutations that had been aligned to the hg38 genome), were then filtered using an awk and grep script to extract the mutations from just the samples in each patient sample group, for downstream analysis.

Script containing awk, grep and sed scripts was used to convert both the MAF and tsv files for each sample group into bed file format, where the columns containing coordinates were reordered to match the correct file format, and chromosome naming was in consistent style. Bedtools (v2.29.2) shuffle was used to shuffle these coordinates to create shuffled controls, excluding ENCODE blacklist regions [75] from hg19, for PCAWG, and hg38 for the TCGA dataset. Bedtools (v2.29.2) window was used, as described above, with options -u -w 5, to find coordinates within 5 bp of predicted G4 sites, predicted non-B DNA sites or shuffled controls. A custom awk script was used to count the occurrence of total mutations, the occurrence of mutations within a 5 bp window of a predicted G4 site, and then the percentage of total mutations within 5 bp of a predicted G4 site. A script using shuf and tail scripts was used to randomly subset the final file containing percentages for all of the samples in the group, to match the number of samples in the SMARCA4 mutant group. To calculate the percentage of patient sample mutations that were within different bins of distance from a predicted G4, bedtools window was used as described above.

### Drug treatment

Pyridostatin hydrochloride (PDS) (SML2690, Sigma-Aldrich) was dissolved in water to obtain a stock solution of 5 mM. BRM014 (MedChemExpress) was dissolved in DMSO to a stock concentration of 10 mM. Cells were treated with the indicated doses of drug, and control cells for BRM014-treated cells were treated with an equivalent concentration of DMSO alone.

### Immunofluorescence

For RPA2 IF, RPE1 parental and *SMARCA4* KO cells were cultured on glass coverslips in 6-well plates until 70% confluency and treated or left untreated with 10 µM PDS for 2 h. Cells were pre-extracted with 0.5% Triton X-100 for 10 min at 4 °C, followed by fixation with 3% (w/v) paraformaldehyde + 2% sucrose in PBS for 10 min at RT. Another fixation step was performed with 100% ice-cold methanol at –20 °C for 10 min. Coverslips were washed five times with PBS and blocked in 3% BSA in PBS—0.01% Tween-20 (PBS-T) for 1 h at RT.

Blocked cells were incubated for 1 h at 37 °C with primary antibodies against RPA2 (mouse Abcam, ab2175, 1:500) and PCNA (rabbit; Abcam, ab18197, 1:500). After three washes with PBS-T, cells were incubated with secondary antibodies (1:2000 dilution): Goat anti-Mouse IgG (H + L) Alexa Fluor 488 (Invitrogen, A-11029, for RPA2) and Donkey anti-Rabbit IgG (H + L) Alexa Fluor 647 (Invitrogen, A-31573, for PCNA), for 1 h at 37 °C. Coverslips were washed three times with PBS-T and mounted with ProLong Gold Antifade Mountant containing DAPI (Thermo Fisher Scientific, P36931), then cured overnight.

For PARylation analysis, cell culture was performed as described above with the following additional steps. Cells were incubated with 10 µM EdU (Invitrogen) for either 15 min prior to PDS treatment (20uM,1 h) or for 30 min during PDS treatment and before fixation. PARGi (Tocris, PDD00017273) was added 20 min before fixation in both conditions. Cells were then pre-extracted for 2 min and processed as described above.

Prior to blocking, coverslips were incubated with the Click-iT™ reaction cocktail containing Alexa Fluor 488 azide for 30 min at room temperature, according to the manufacturer’s instructions (Invitrogen, C10637). After EdU detection and blocking, cells were incubated with anti-poly(ADP-ribose) mouse monoclonal antibody (10H; ENZO, ALX-804–220, 1:250) for 1 h at 37 °C, followed by three washes with PBS-T. Coverslips were then incubated with Donkey anti-Rabbit IgG (H + L) Alexa Fluor 647 (Invitrogen, A-31573, 1:2000) for 1 h at 37 °C, washed, and mounted as described above.

For yH2AX IF, 200,000 RPE1 or *SMARCA4* KO cells were seeded on coverslips in 6-well plates and grown overnight. Cells were treated with 20 μM PDS for 3 or 6 h. Cells were fixed with 4% paraformaldehyde for 15 min, permeabilised with 0.5% Triton X-100 for 8 min, and blocked with 1% BSA for 30 min. Coverslips were incubated in γH2A.X rabbit polyclonal antibody (Cell Signalling Technology, #2577, 1:1000) at 4 °C overnight, and in Alexa Fluor 488-conjugated Donkey anti-Rabbit secondary antibody (Invitrogen, A-21206, 1:1000) at room temperature for 1 h. Slides were mounted with VECTASHIELD Antifade mounting media with DAPI.

Imaging was performed using a CSU-W1 Yokogawa Advanced Spinning Disk (PARylation) or CSU-W1 SoRa Yokogawa Super-Resolution Spinning Disk Confocal microscope (RPA2), or Zeiss Axio Observer Z1 Marianas Microscope (yH2AX) equipped with SlideBook software (3i). Z-stacks were acquired at 1 µm intervals using a 63 × oil objective, or 40 × for yH2AX, and images were exported as maximum intensity projections for analysis. Foci number, intensity, and nuclear intensity were quantified from three independent biological replicates using CellProfiler (v4.0.7). Statistical analyses were performed in GraphPad Prism v10.6.1.

### Sulforhodamine B (SRB) assay

Cells were seeded in triplicate into 96-well plates at a density of 1,000 cells/well, and allowed to attach and grow for 24 h. PDS and/or BRM014, or the equivalent concentration of DMSO alone, was then added and cells were incubated for 6 days in total. After this, cells were fixed by the addition of 100 µl 10% TCA (Merck) and stored at 4 °C for at least 24 h. Plates were washed five times with water and allowed to completely dry before staining with 0.57% SRB powder (Merck) dissolved in 1% acetic acid and incubated for 1 h at RT. Unbound dye was removed by washing with 1% acetic acid, and the plate was allowed to dry completely. Protein-bound dye was solubilised in 10 mM Tris–HCl, pH = 9.5 for 30 min at room temperature with gentle rocking. Absorbance at 565 nm was quantified using the SpectraMax Microplate Absorbance Reader (Perkin Elmer). The average of three technical replicates was used as a single datapoint for each biological replicate, and survival was defined as a percentage of the absorbance measured in treated wells compared with control (DMSO-only) wells.

### Cell death (Celigo) assay

Cells were seeded in triplicate into black, clear-bottom 96-well plates (Greiner Bio-One) at a density of 1,000 cells/well and were allowed to attach and grow for 24 h. Cells were treated with the indicated compounds for 5 days. Propidium iodide (Merck) and Hoechst (ThermoFisher Scientific) were added to wells to a final concentration of 2 µg/mL each, and cells were incubated at RT for 30 min, protected from light. Total (Hoechst-positive) and dead (PI-positive) nuclei were detected using a Celigo S image cytometer (Revvity) and were used to determine the % of dead cells in the population.

### Protein extracts and western blotting

Cell pellets were lysed in 50 mM Tris pH 7.9, 8 M Urea, 1%Chaps and incubated at 4 °C with agitation for at least 30 min. The lysate was cleared by centrifugation and the supernatant was collected. The protein concentration of the extracts was measured by Bradford assay and 25 μg of supernatants were resolved by SDS–polyacrylamide gel electrophoresis and transferred onto a Hybond-C Extra Nitrocellulose membrane (Fisher Scientific UK, Loughborough, UK). The membrane was blocked in 5% milk, 0.1% Tween-20 in TBS buffer for 1 h and probed overnight with antibodies against SMARCA4 (BRG1) (Santa Cruz, sc-17796, dilution 1:2000), a-tubulin (Abcam Ab7291, dilution 1:5000) in 5% milk, 0.1% Tween-20 in TBS buffer. The membrane was washed three times with 0.1% Tween-20 in TBS and incubated for 1 h with horseradish peroxidase (HRP)-conjugated secondary antibodies in 5% milk, 0.1% Tween-20 in TBS. The secondary antibodies used were rabbit anti-mouse HRP (Dako, P0260, dilution 1:5000) and goat anti-rabbit HRP (Dako, P0448, dilution 1:5000). Proteins were visualised by using in-house ECL reagent or SuperSignal West Pico Chemiluminescent Substrate (Life Technologies).

### Chromatome cell preparation

Parental RPE1 and *SMARCA4* KO1 cells were seeded into 15-cm plates (four plates for each condition) and incubated until they reached 80–90% confluency. The day of the treatment, media was replaced with new media containing PDS (20 uM or 1uM) or not. Cells were incubated for 4 h at 37 °C before collection after trypsin detachment. Cells were washed with PBS, snap frozen and stored at −70 °C.

### Chromatin enrichment

Flash frozen cell pellets were thawed on ice and resuspended in nuclear extraction buffer (15 mM Tris–HCl pH7.5, 60 mM KCl, 15 mM NaCl, 5 mM MgCl2, 1 mM CaCl2, 250 mM sucrose, 0.3% NP-40, freshly supplemented with 1 mM DTT and Halt Protease and Phosphatase inhibitor (Thermo Scientific) and incubated on ice for 5 min. Nuclei were collected by centrifugation (600 rcf, 5 min at 4 °C), washed once with nuclear extraction buffer without NP-40 pelleted again, then re-suspended in pre-chilled hypotonic buffer (3 mM EDTA, 0.2 mM EGTA and freshly supplemented with 1 mM DTT and Halt Protease and Phosphatase inhibitor) and incubated on ice for 30 min to release chromatin. Chromatin was pelleted for 5 min at 1,700 rcf at 4 °C cooled centrifuge and subsequently washed twice with hypotonic buffer.

### MS sample preparation and TMT labelling

Chromatin pellets were solubilised using probe sonication in lysis buffer 100 mM triethylammonium bicarbonate (TEAB), 1% sodium deoxycholate (SDC), 10% isopropanol, 50 mM NaCl, 1:1,000 Pierce Universal Nuclease (Thermo Scientific) supplemented with Halt Protease and Phosphatase inhibitor. Protein concentration was measured with the Quick Start Bradford protein assay (Bio-Rad) following the manufacturer’s protocol. Protein with an equal contribution from each individual sample were reduced with 5 mM tris-2-carboxyethyl phosphine (TCEP) for 1 h, followed by alkylation with 10 mM iodoacetamide (IAA) for 30 min, then digested by adding trypsin (Pierce) at final concentration 75 ng/μL to each sample and incubating the samples for 18 h at RT. For each sample, 30 μg was allocated for chromatin proteomics and peptides were labelled with TMTpro multiplexing reagents (Thermo Scientific) according to the manufacturer’s protocol. Once the samples were combined, SDC was precipitated by adding formic acid (FA) to a final concentration of 2% (v/v), followed by centrifugation at 10,000 rpm for 5 min. The supernatant containing TMT-labelled peptides was dried using a centrifugal vacuum concentrator.

### High-pH reversed-phase peptide fractionation and liquid chromatography–mass spectrometry analysis

Prior to MS, TMT-labelled peptides were fractionated with high-pH reversed-phase (RP) chromatography using the Waters XBridge C18 column (2.1 × 150 mm, 3.5 μm) on a Dionex UltiMate 3000 high-performance liquid chromatography (HPLC) system. Mobile phase A was 0.1% ammonium hydroxide (v/v), mobile phase B was 100% acetonitrile and 0.1% ammonium hydroxide (v/v). Peptide separation was performed with a gradient elution at 200 μL/min with the following steps: isocratic for 5 min at 5% phase B, gradient for 40 min to 35% phase B, gradient to 80% phase B in 5 min, isocratic for 5 min, and re-equilibrated to 5% phase B. Fractions were collected in a 96-well plate every 42 s to a total of 65 fractions, then concatenated into 12 fractions, dried and reconstituted in 0.1% TFA.

### LC–MS analysis

LC–MS analysis was performed on the Dionex UltiMate 3000 UHPLC system coupled with Orbitrap Ascend mass spectrometers (Thermo Scientific). Samples were analysed with the EASY-Spray C18 capillary column (75 μm × 50 cm, 2 μm) at 50 °C. Mobile phase A was 0.1% formic acid and mobile phase B was 80% acetonitrile, 0.1% formic acid. The gradient separation method was as follows: 150 min gradient up to 38% B, for 10 min up to 95% B, for 5 min isocratic at 95% B, re-equilibration to 5% B in 10 min, for 10 min isocratic at 5% B. Precursors between 375 and 1500 m/z were selected with a mass resolution of 120 000, automatic gain control (AGC) of 4 × 105, and IT (injection time) of 50 ms, with the top speed mode in 3 s for high collision dissociation (HCD) fragmentation with a quadrupole isolation width of 0.7 Th (Thomson unit). The collision energy was set at 35% (TMTpro) with AGC at 1 × 105 and IT at 86 ms. A Dionex Ultimate 3000 system and mass spectrometer (Thermo Scientific) were used for data acquisition. From each fraction 10 μL was injected onto a C18 trapping column (Acclaim PepMap 100, 100 μm × 2 cm, 5 μm, 100 Å) at a 10 μL/min flow rate. The samples were subjected to a low-pH gradient elution on a nanocapillary reversed phase column (Acclaim PepMap C18, 75 μm × 50 cm, 2 μm, 100 Å) at 45 °C. Mobile phases A and B were 0.1% formic acid and 80% acetonitrile, 0.1% formic acid respectively. The separation was performed at 300 nL/min flow rate and 90 min gradient from 5 to 38% phase B followed by 10 min up to 95% phase B, isocratic for 5 min at 95% B, re-equilibrated to 5% phase B in 5 min, and isocratic for 10 min at 5% phase B. MS1 scans were collected with mass resolution of 120,000, automatic gain control of 4 × 105, and injection time of 50 ms. MS2 spectra were acquired in the ion trap using a Turbo scan rate, with 32% HCD collision energy and a maximum injection time of 35 ms. Real-time database searching against canonical Homo sapiens database was conducted using the Comet search engine, considering tryptic peptides with a maximum of 2 missed cleavage. Static modifications were set for carbamidomethylation of C (+ 57.0215 Da) and TMTpro labelling on K and N-termini (+ 304.207 Da). Variable modifications included deamidation of N/Q (+ 0.984 Da) and oxidation of M (+ 15.9949 Da), allowing up to 2 variable modifications per peptide. A close-out feature was enabled, limiting to 4 peptides per protein. SPS10-MS3 scans were performed on selected precursors using the Orbitrap at 45,000 resolution with 35% HCD collision energy, a 200% normalized AGC target, and a 200 ms maximum injection time. Data were collected in centroid mode with single micro-scan acquisition.

### Database search and protein quantification

The SequestHT and Comet search engines were used to analyse the acquired spectra in Proteome Discoverer 3.0 (Thermo Scientific) for protein identification and quantification. The precursor mass was set to 20 ppm and fragment mass tolerance was 0.5 Da. Spectra were searched for fully tryptic peptides with maximum 2 missed-cleavages. TMTpro at N-term/Lys and Carbamidomethyl at Cys were defined as static modifications. Dynamic modifications included oxidation of Met and Deamidation of Asn/Gln. Peptide confidence was estimated with the Percolator node. Peptide FDR was set at 1% and validation was based on q-value and target-decoy database search. Spectra were searched against reviewed UniProt human protein entries. The reporter ion quantifier node included a TMT quantification method with an integration window tolerance of 15 ppm and integration method based on the most confident centroid peak at the MS3. Only unique peptides were used for quantification, considering protein groups for peptide uniqueness. Peptides with average reporter signal-to-noise > 3 were used for quantification. The data was median normalised at the proteome level. Relative abundances were calculated by dividing normalised protein/peptide abundances by the average abundance of all TMT channels per biological replicate. PRIDE Accession code is PXD065273.

### Quantification and statistical analysis

Depending on the experiment and the appropriate statistical test, two-way ANOVA with Tukey’s or Šídák's multiple comparisons test, Kruskal–Wallis test with multiple comparisons, one-way ANOVA with Kruskal–Wallis test, or one-sample Wilcoxon test of the medians were used, and the analysis used is indicated in the corresponding figure legend for each experiment. Graphs were generated and statistical analyses performed using GraphPad Prism (v10.6.1) or R (v4.3.1) in RStudio (2024.12.0). Microscopy images were analysed using ImageJ (v1.5.3 or 1.5.4), CellProfiler (v4.0.7) or MetaXpress software (v6.7.2.290), and visualized using GraphPad Prism. Analysis of omics datasets was performed using the CentOS8 linux distribution platform (based on Redhat Enterprise Linux Operating System), on a high-performance computer with 1248 × Intel Xeon Platinum 8260 (Cascade Lake) @ 2.40 GHz. Figures were generated using Inkscape software. Some graphics were generated using BioRender (biorender.com), indicated in figure legends.

## Supplementary Information

Additional file 1: Figs. S1 – S10.

Additional file 2: Figs. S1 – S5. Original, uncropped images of all gels and blots.

## Data Availability

ATAC-seq datasets generated in this work are available in the NCBI gene expression omnibus (GEO) under accession code GSE308091 [76]. INDUCE-seq and WGS datasets have been deposited in the NCBI sequence read archive (SRA) under accession number PRJNA1331556 [77]. The chromatin bound mass spectrometry dataset is available in PRIDE under accession code PXD065273 [78]. The scripts and packages used are described in the methods and source code is available at Github (https://github.com/Downs-Lab/SMARCA4_G4) [79] and Zenodo (10.5281/zenodo.19471554) [80]. Previously deposited datasets used in this study include SMARCA4 CUT& RUN (GSE235294) [81] as described in [54], G4 mapping data (GSE178668) [82] as described in [32], and histone mark ChIP-seq data (GSE175752) [83] from [67]. Cancer patient data was obtained from PCAWG [46] and TCGA SKCM [47] and accessed via cBioportal [74].
