# Supplementary material for: The SMARCA4 subunit of the SWI/SNF complex prevents genome instability at G quadruplexes
Source: Genome Biol. 2026 Apr 20;27:180. doi: 10.1186/s13059-026-04080-4 (PMC13224610; doi:10.1186/s13059-026-04080-4)
Supplement: Supplementary file 1 — Additional file 1: Figs. S1 – S10. [file 13059_2026_4080_MOESM1_ESM.pdf]

Fig S1

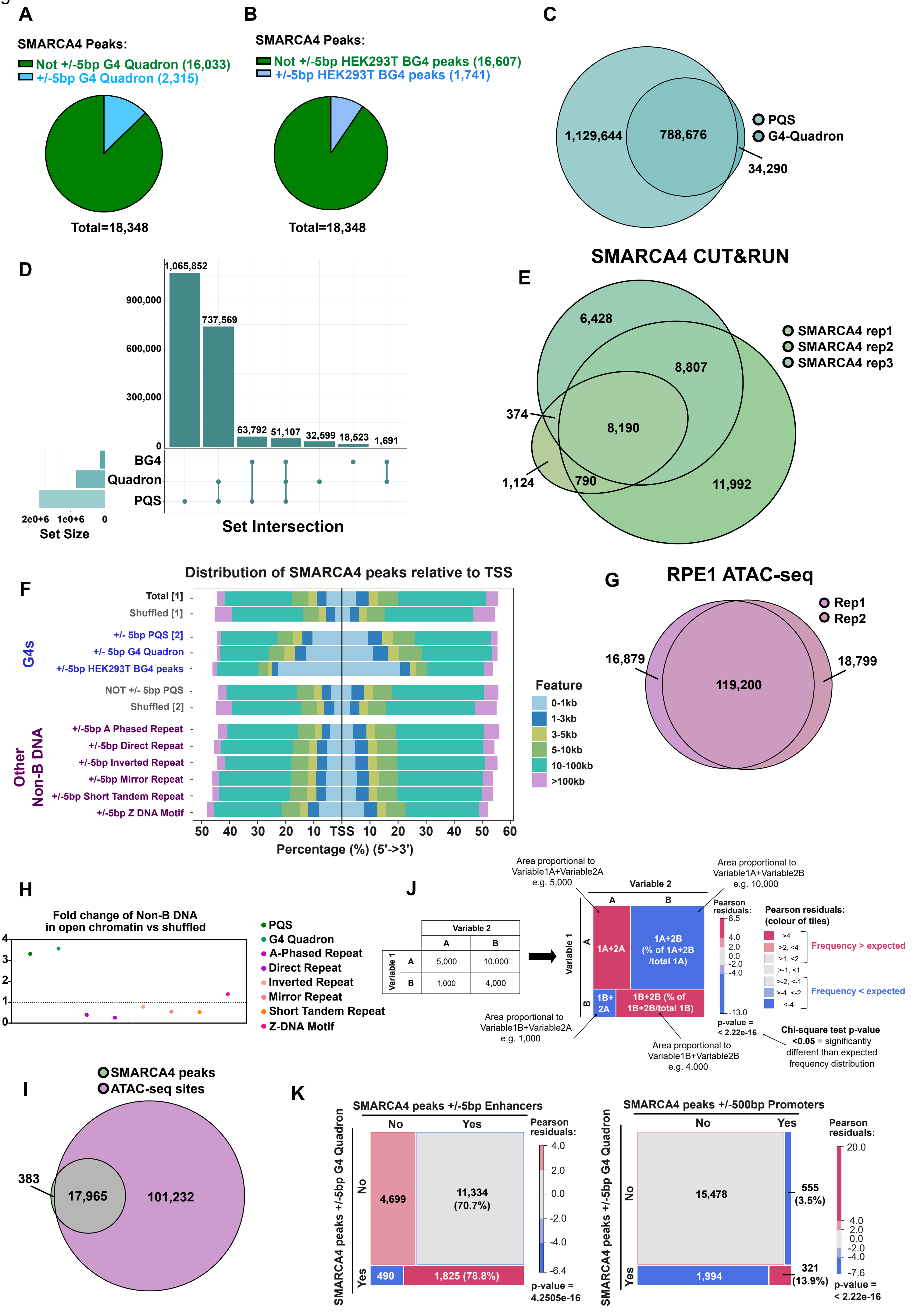

A
SMARCA4 Peaks:
Not +/-5bp G4 Quadron (16,033)
+/-5bp G4 Quadron (2,315)
Total=18,348
B
SMARCA4 Peaks:
Not +/-5bp HEK293T BG4 peaks (16,607)
+/-5bp HEK293T BG4 peaks (1,741)
Total=18,348
C
1,129,644
788,676
34,290
PQS
G4-Quadron
D
1,065,852
737,569
63,792
51,107
32,599
18,523
1,691
900,000
600,000
300,000
0
BG4
Quadron
PQS
2e0+6
1e0+6
0
Set Size
Set Intersection
SMARCA4 CUT&RUN
E
6,428
8,807
374
8,190
1,124
790
11,992
SMARCA4 rep1
SMARCA4 rep2
SMARCA4 rep3
F
Distribution of SMARCA4 peaks relative to TSS
Total [1]
Shuffled [1]
G4s
+/- 5bp PQS [2]
+/- 5bp G4 Quadron
+/-5bp HEK293T BG4 peaks
NOT +/- 5bp PQS
Shuffled [2]
Other Non-B DNA
+/-5bp A Phased Repeat
+/-5bp Direct Repeat
+/-5bp Inverted Repeat
+/-5bp Mirror Repeat
+/-5bp Short Tandem Repeat
+/-5bp Z DNA Motif
50 40 30 20 10 TSS 10 20 30 40 50 60
Percentage (%) (5'->3')
Feature
0-1kb
1-3kb
3-5kb
5-10kb
10-100kb
>100kb
G
RPE1 ATAC-seq
Rep1
Rep2
16,879
119,200
18,799
H
Fold change of Non-B DNA in open chromatin vs shuffled
4
3
2
1
0
PQS
G4 Quadron
A-Phased Repeat
Direct Repeat
Inverted Repeat
Mirror Repeat
Short Tandem Repeat
Z-DNA Motif
J
Variable 2
A
B
Variable 1
A
5,000
10,000
B
1,000
4,000
Area proportional to Variable1A+Variable2A e.g. 5,000
Area proportional to Variable1A+Variable2B e.g. 10,000
1A+2A
1A+2B (% of 1A+2B /total 1A)
1B+2A
1B+2B (% of 1B+2B/total 1B)
Pearson residuals:
8.5
4.0
2.0
0.0
-2.0
-4.0
-13.0
p-value = < 2.22e-16
Area proportional to Variable1B+Variable2A e.g. 1,000
Area proportional to Variable1B+Variable2B e.g. 4,000
Pearson residuals: (colour of tiles)
>4
>2, <4
>1, <2
>-1, <1
>-2, <-1
>-4, <-2
<-4
Frequency > expected
Frequency < expected
Chi-square test p-value <0.05 = significantly different than expected frequency distribution
I
SMARCA4 peaks
ATAC-seq sites
383
17,965
101,232
K
SMARCA4 peaks +/-5bp Enhancers
No
Yes
SMARCA4 peaks +/-5bp G4 Quadron
4,699
11,334 (70.7%)
490
1,825 (78.8%)
Pearson residuals:
4.0
2.0
0.0
-2.0
-4.0
-6.4
p-value = 4.2505e-16
SMARCA4 peaks +/-500bp Promoters
15,478
555 (3.5%)
1,994
321 (13.9%)
Pearson residuals:
20.0
4.0
2.0
0.0
-2.0
-4.0
-7.6
p-value = < 2.22e-16

Fig S1. SMARCA4 binds at predicted G4 sites with features associated with open chromatin.

A-B Pie chart representing the proportion of SMARCA4 peaks (significant in at least 2 replicates) that are within 5bp of G4 Quadron sites (**A**) or significant BG4 peaks (**B**). **C** Venn diagram indicating overlap of PQS and G4-Quadron sites. **D** Upset plot indicating overlap of PQS, G4-Quadron and significant BG4 peaks. **E** Venn diagram indicating the overlap of significant SMARCA4 peaks in CUT&RUN replicates. **F** Stacked colour bar representing the genomic distribution of significant SMARCA4 peaks, by relative distance to TSS. SMARCA4 peaks are defined either as total (significant in at least 2 replicates), those within a window of 5bp of either predicted G4s (PQS, G4-Quadron, BG4 peaks) or alternative Non-B DNA motifs, those not within a window of 5bp of PQS, or shuffled controls where the coordinates of these peak groups (specified in square brackets) are shuffled around the genome. **G** Venn diagram indicating the overlap of significant ATAC-seq replicates. **H** Dot plot representing the fold change of Non-B DNA motifs that are within 5bp of open chromatin regions (defined by significant ATAC-seq peaks in both replicates) versus a shuffled control. **I** Venn diagram indicating the overlap of significant SMARCA4 peaks (found in at least 2 replicates) with open chromatin (significant in both reps of ATAC-seq). **J** Schematic to explain the interpretation of mosaic plots. + indicates cooccurrence. **K** Mosaic plots of observed frequencies of cooccurrence of SMARCA4 consensus peaks with G4 Quadron (within 5bp window) and either enhancers (within 5bp window, top) or promoters (within 500bp window, bottom). Numbers of SMARCA4 peaks in each subset are annotated, with percentages of peaks in each row that cooccur with enhancers or promoters, respectively. Tile sizes are proportional to the observed frequencies of 2-way subsets. Red indicates positive and blue indicates negative Pearson residuals from chi-square test. P-value indicates evidence for significant difference from expected values.

Fig S2

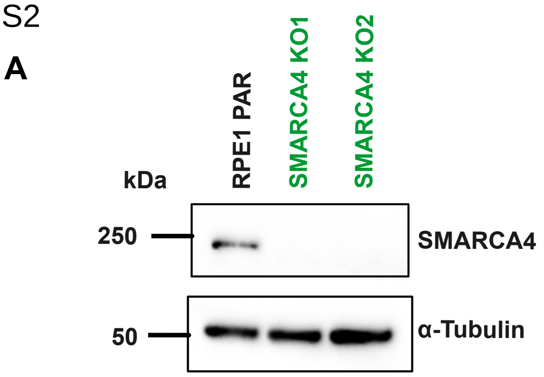

B

	DSBs	DSBs +/- 5bp PQS	DSBs +/- 5bp Quadron
RPE1 PAR_1	207,616	4,398	1,905
RPE1 PAR_2	280,221	5,863	2,490
SMARCA4 KO1_1	238,806	5,505	2,501
SMARCA4 KO1_2	302,532	6,626	2,905
SMARCA4 KO2_1	281,874	6,833	3,079
SMARCA4 KO2_2	456,999	10,658	4,642

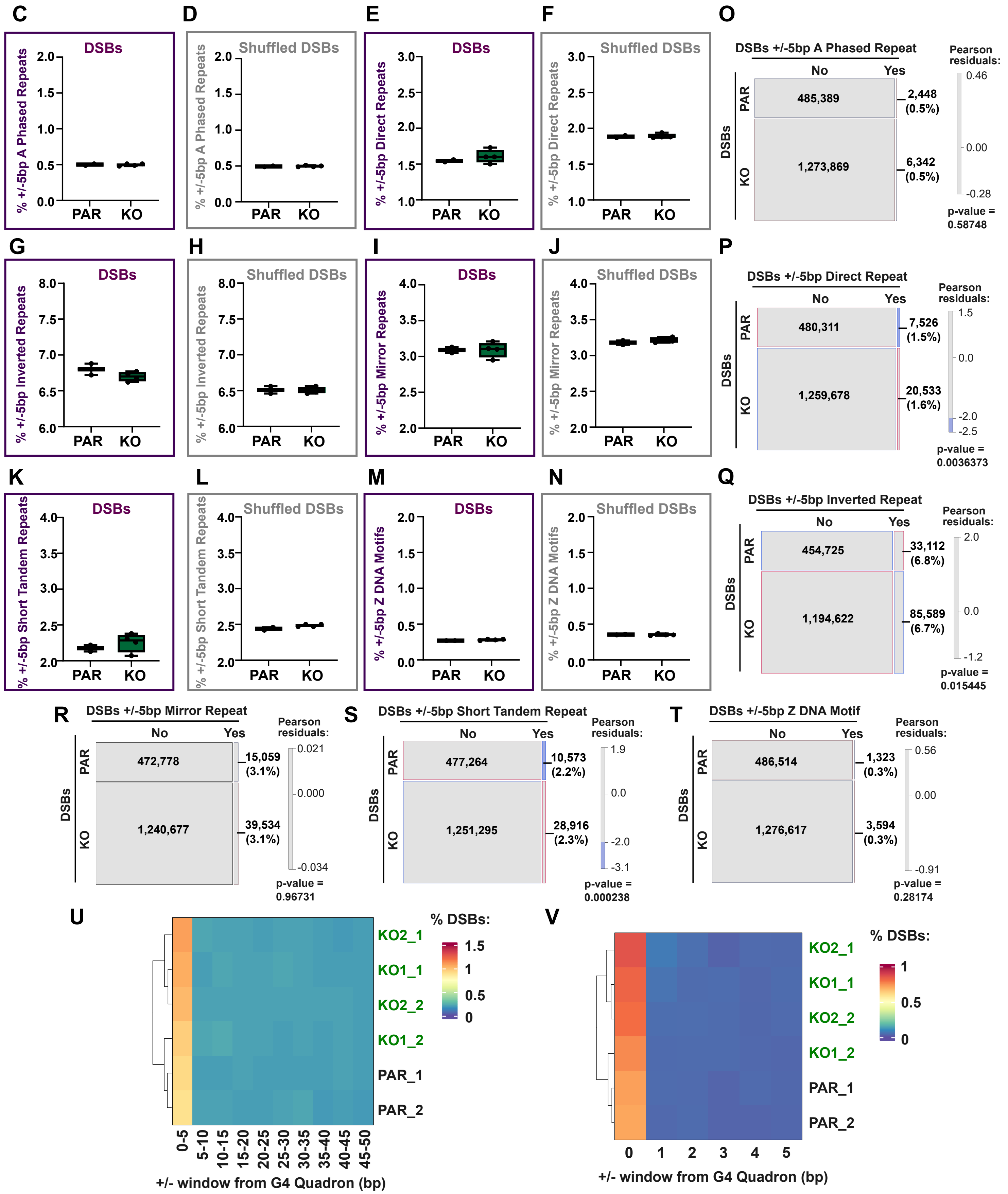

Fig S2. DSBs are more likely near predicted G4s than non-B DNA motifs in absence of SMARCA4.

A Western blotting of whole cell lysates from parental and SMARCA4 KO cells (clones KO1 and KO2) for SMARCA4. α-tubulin is used as a loading control. Full uncropped blots in Additional file 2: Fig S1. **B** Table containing the total number of DSBs detected per replicate, and the number within a 5bp window of PQS or G4 Quadron sites. **C-N** Box and whisker plots indicating percentage of DSBs within 5bp window of Non-B DNA motif sites, grouped by parental replicates (PAR) and SMARCA4 KO replicates for both clones (KO), with replicates plotted as individual points. Box and whisker plots represent total DSBs (**C,E,G,I,K,M**) or shuffled DSBs, as described in Figure **1F** (**D,F,H,J,L,N**). **O-T** Mosaic plots of observed frequencies of cooccurrence of DSBs (PAR (parental, replicates combined), KO (SMARCA4 KO, replicates and KO clones combined)) with non-B DNA motifs (within 5bp window). Numbers of DSBs in each subset are annotated, with percentages of DSBs in each row that cooccur with the non-B DNA motifs. Tile sizes are proportional to the observed frequencies. Red indicates positive and blue indicates negative Pearson residuals from chi-square test. Overall p-value indicates evidence for significant difference from expected values. **U-V** Hierarchical heatmaps representing the percentage of DSBs that are within a window of G4 Quadron sites, either shown in 5bp bins from 0-50bp (**U**) or in 1bp bins from 0-5bp (**V**).

Fig S3

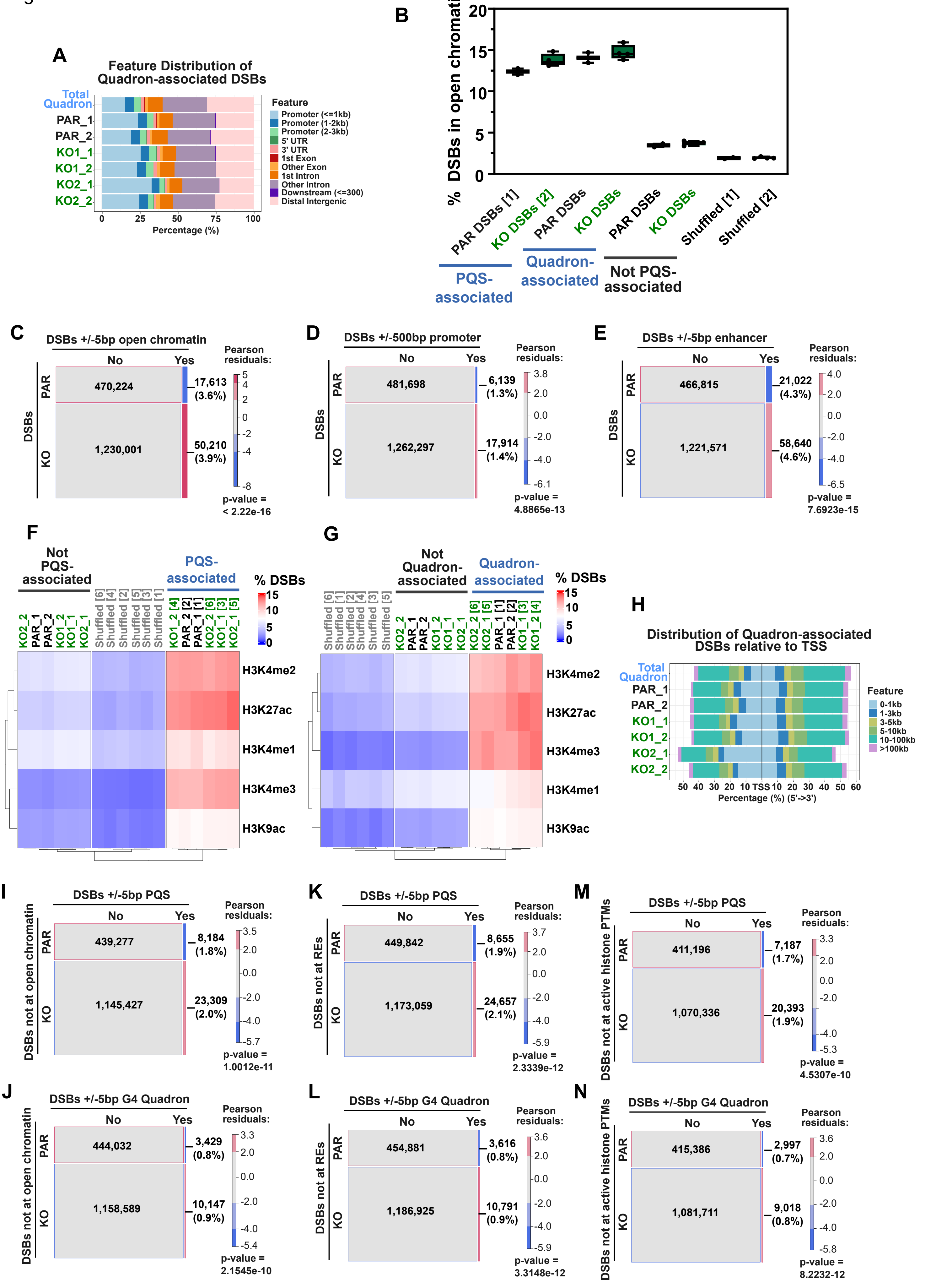

A
Feature Distribution of Quadron-associated DSBs
Total Quadron
PAR_1
PAR_2
KO1_1
KO1_2
KO2_1
KO2_2
Percentage (%)
Feature
Promoter (<=1kb)
Promoter (1-2kb)
Promoter (2-3kb)
5' UTR
3' UTR
1st Exon
Other Exon
1st Intron
Other Intron
Downstream (<=300)
Distal Intergenic
B
% DSBs in open chromatin
PAR DSBs [1]
KO DSBs [2]
PAR DSBs
KO DSBs
PAR DSBs
KO DSBs
Shuffled [1]
Shuffled [2]
PQS-associated
Quadron-associated
Not PQS-associated
C
DSBs +/-5bp open chromatin
No Yes
PAR 470,224 17,613 (3.6%)
KO 1,230,001 50,210 (3.9%)
Pearson residuals:
p-value = < 2.22e-16
D
DSBs +/-500bp promoter
PAR 481,698 6,139 (1.3%)
KO 1,262,297 17,914 (1.4%)
p-value = 4.8865e-13
E
DSBs +/-5bp enhancer
PAR 466,815 21,022 (4.3%)
KO 1,221,571 58,640 (4.6%)
p-value = 7.6923e-15
F
Not PQS-associated
PQS-associated
% DSBs
H3K4me2
H3K27ac
H3K4me1
H3K4me3
H3K9ac
G
Not Quadron-associated
Quadron-associated
H
Distribution of Quadron-associated DSBs relative to TSS
Percentage (%) (5'->3')
0-1kb
1-3kb
3-5kb
5-10kb
10-100kb
>100kb
I
DSBs +/-5bp PQS
DSBs not at open chromatin
PAR 439,277 8,184 (1.8%)
KO 1,145,427 23,309 (2.0%)
p-value = 1.0012e-11
K
DSBs +/-5bp PQS
DSBs not at REs
PAR 449,842 8,655 (1.9%)
KO 1,173,059 24,657 (2.1%)
p-value = 2.3339e-12
M
DSBs +/-5bp PQS
DSBs not at active histone PTMs
PAR 411,196 7,187 (1.7%)
KO 1,070,336 20,393 (1.9%)
p-value = 4.5307e-10
J
DSBs +/-5bp G4 Quadron
DSBs not at open chromatin
PAR 444,032 3,429 (0.8%)
KO 1,158,589 10,147 (0.9%)
p-value = 2.1545e-10
L
DSBs +/-5bp G4 Quadron
DSBs not at REs
PAR 454,881 3,616 (0.8%)
KO 1,186,925 10,791 (0.9%)
p-value = 3.3148e-12
N
DSBs +/-5bp G4 Quadron
DSBs not at active histone PTMs
PAR 415,386 2,997 (0.7%)
KO 1,081,711 9,018 (0.8%)
p-value = 8.2232-12

Fig S3. DSBs near predicted G4s more likely to occur at sites associated with open chromatin.

A Stacked colour bar charts representing genomic distribution of DSBs within 5bp of G4 Quadron sites, categorised by feature. Distribution of total G4 Quadron sites is plotted. **B** Box and whisker plot indicating percentage of DSBs within open chromatin, i.e. within 5bp of significant ATAC-seq peaks, identified in both replicates. DSBs are defined as total, PQS-associated or Quadron-associated, Not PQS-associated, or shuffled controls (of groups specified in square brackets). **C-E** Mosaic plots of observed frequencies of cooccurrence of DSBs (replicates and KO clones combined) with either open chromatin (**C**), promoters (**D**, within 500bp) or enhancers (**E**, within 5bp). Numbers of DSBs in each subset are annotated, with percentages of DSBs in each row that cooccur with open chromatin, promoters or enhancers. **F-G** Hierarchical heatmaps representing the percentage of DSBs within 5bp of significant histone PTM peaks, either for PQS-associated DSBs (**F**) or Quadron-associated DSBs (**G**). Shuffled controls are also shown (of groups specified in square brackets). **H** Stacked colour bar chart representing genomic distribution of DSBs within 5bp of G4 Quadron sites, categorised by relative distance to TSS. Distribution of total G4 Quadron sites is plotted. **I-N** Mosaic plots of observed frequencies of cooccurrence of DSBs not at potential covariate sites, with either PQS (**I,K,M**) or G4 Quadron sites (**J,L,N**). Covariate sites, which overlapped with DSBs and were excluded from this analysis, were defined as either open chromatin (**I,J**, DSBs within 500bp of ATAC-seq peaks, identified in both replicates), regulatory elements (REs, **K**,**L**, DSBs within 500bp of promoters or enhancers), or active histone PTMs (**M**,**N**), DSBs within 500bp of H3K27ac, H3K4me1, H3K4me2, H3K4me3 or H3K9ac significant peaks. Numbers of DSBs in each subset are annotated, with percentages of DSBs in each row that cooccur with either PQS or G4 Quadron sites. For **C-E** and **I-N**, tile sizes are proportional to observed frequencies. Red indicates positive and blue indicates negative Pearson residuals from chi-square test. P-value indicates evidence for significant difference from expected values.

Fig S4

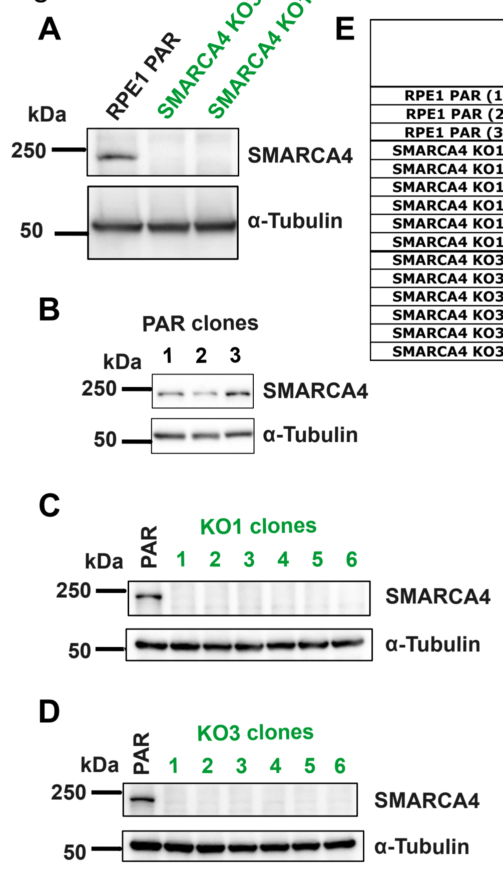

E

	SNVs	Deletions	Insertions	SNVs +/-5bp PQS	Deletions +/-5bp PQS	Insertions +/-5bp PQS	SNVs +/-5bp Quadron	Deletions +/-5bp Quadron	Insertions +/-5bp Quadron
RPE1 PAR (1)	1,354	32	28	33	0	1	12	0	0
RPE1 PAR (2)	1,368	42	26	44	1	3	16	1	0
RPE1 PAR (3)	1,348	34	35	34	0	0	13	0	0
SMARCA4 KO1 (1)	1,244	26	15	35	1	0	14	1	0
SMARCA4 KO1 (2)	1,082	33	17	35	1	0	11	0	0
SMARCA4 KO1 (3)	1,529	25	11	48	1	0	19	0	0
SMARCA4 KO1 (4)	1,237	35	14	45	2	0	22	2	0
SMARCA4 KO1 (5)	1,176	40	17	44	2	0	18	1	0
SMARCA4 KO1 (6)	1,006	21	14	32	1	1	15	1	0
SMARCA4 KO3 (1)	2,261	50	23	79	1	0	28	0	0
SMARCA4 KO3 (2)	1,203	45	26	44	1	0	22	0	0
SMARCA4 KO3 (3)	1,172	32	33	51	2	0	19	1	0
SMARCA4 KO3 (4)	1,229	29	17	29	2	0	19	0	0
SMARCA4 KO3 (5)	1,133	27	26	36	1	1	17	0	0
SMARCA4 KO3 (6)	1,260	30	29	47	1	0	17	0	0

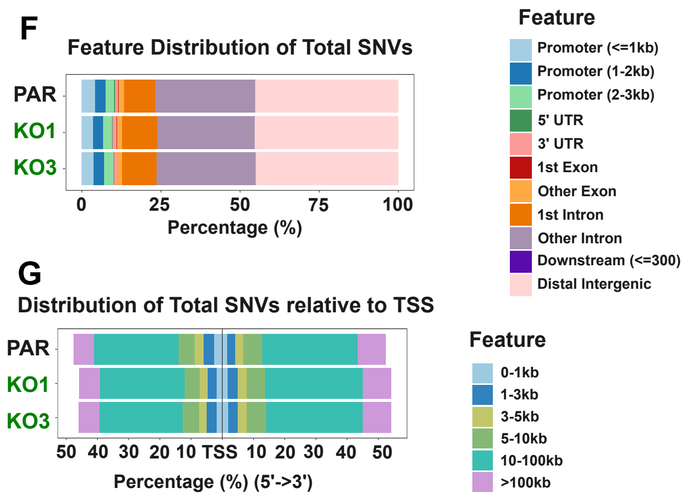

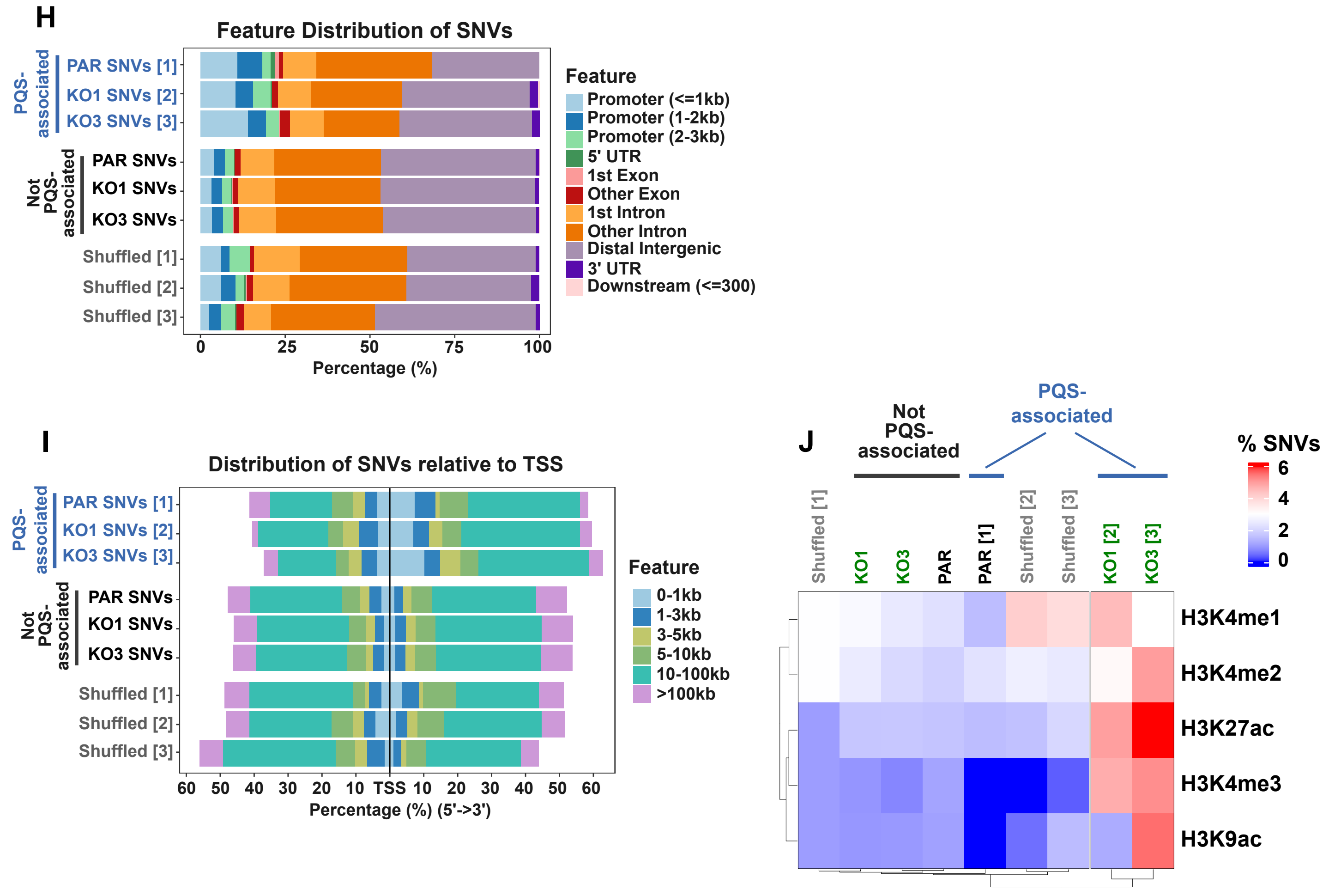

Fig S4. SNVs near predicted G4s more likely to occur in open chromatin, in absence of SMARCA4.

A-D Western blotting of whole cell lysates from parental and SMARCA4 KO cells for SMARCA4, for clones KO1 and KO3 (**A**), and for single-cell sorted clones following 1 month of culture for parental clones 1-3 (**B**), for KO1 clones 1-6 (**C**) and KO3 clones 1-6 (**D**). α-tubulin is used as a loading control. Full uncropped blots for **A-D** in Additional file 2: Fig S2-5. **E** Table containing the total number of SNVs, deletions and insertions detected per clones (indicated in brackets), and the number within a 5bp window of PQS or G4 Quadron sites. **F-G** Stacked colour bars representing the genomic distribution of total SNVs, either categorised by feature (**F**) or relative distance to TSS (**G**). **H-I** Stacked colour bars representing the genomic distribution of SNVs that are either PQS-associated (those within a 5bp window of PQS, labelled in blue), Not PQS-associated (those that are not within a 5bp window of PQS, labelled in black), or a shuffled control, where the coordinates of these peak groups (specified in square brackets) have been shuffled around the genome (labelled in grey), either categorised by feature (**H**) or relative distance to TSS (**I**). **J** Hierarchical heatmap representing the percentage of SNVs within a 5bp window of significant active histone PTM peaks, PQS-associated, not PQS-associated, and shuffled controls, as defined in part **H-I**.

Fig S5

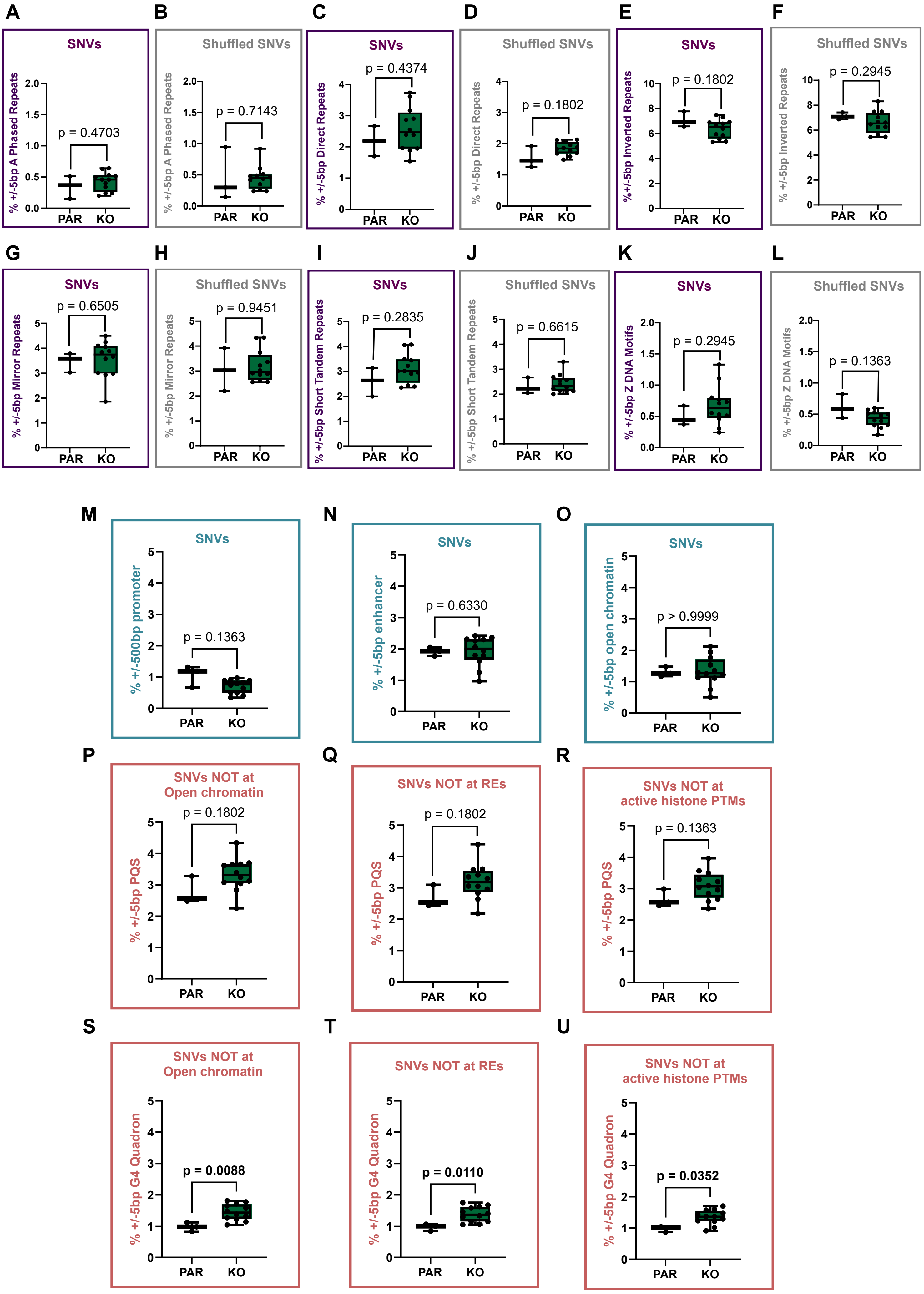

A
SNVs
% +/-5bp A Phased Repeats
p = 0.4703
PAR
KO
B
Shuffled SNVs
% +/-5bp A Phased Repeats
p = 0.7143
PAR
KO
C
SNVs
% +/-5bp Direct Repeats
p = 0.4374
PAR
KO
D
Shuffled SNVs
% +/-5bp Direct Repeats
p = 0.1802
PAR
KO
E
SNVs
%+/-5bp Inverted Repeats
p = 0.1802
PAR
KO
F
Shuffled SNVs
% +/-5bp Inverted Repeats
p = 0.2945
PAR
KO
G
SNVs
% +/-5bp Mirror Repeats
p = 0.6505
PAR
KO
H
Shuffled SNVs
% +/-5bp Mirror Repeats
p = 0.9451
PAR
KO
I
SNVs
% +/-5bp Short Tandem Repeats
p = 0.2835
PAR
KO
J
Shuffled SNVs
% +/-5bp Short Tandem Repeats
p = 0.6615
PAR
KO
K
SNVs
% +/-5bp Z DNA Motifs
p = 0.2945
PAR
KO
L
Shuffled SNVs
% +/-5bp Z DNA Motifs
p = 0.1363
PAR
KO
M
SNVs
% +/-500bp promoter
p = 0.1363
PAR
KO
N
SNVs
% +/-5bp enhancer
p = 0.6330
PAR
KO
O
SNVs
% +/-5bp open chromatin
p > 0.9999
PAR
KO
P
SNVs NOT at
Open chromatin
% +/-5bp PQS
p = 0.1802
PAR
KO
Q
SNVs NOT at REs
% +/-5bp PQS
p = 0.1802
PAR
KO
R
SNVs NOT at
active histone PTMs
% +/-5bp PQS
p = 0.1363
PAR
KO
S
SNVs NOT at
Open chromatin
% +/-5bp G4 Quadron
p = 0.0088
PAR
KO
T
SNVs NOT at REs
% +/-5bp G4 Quadron
p = 0.0110
PAR
KO
U
SNVs NOT at
active histone PTMs
% +/-5bp G4 Quadron
p = 0.0352
PAR
KO

Fig S5. SNVs more likely near predicted G4s, but not other non-B motifs in absence of SMARCA4.

A-L Box and whisker plots indicating percentage of SNVs within a 5bp window of predicted Non-B DNA sites, grouped by parental clones (PAR) and SMARCA4 KO clones (KO), with clones plotted as individual points. Box and whisker plots represent the percentage of SNVs (**A,C,E,G,I,K**) or the percentage of shuffled coordinates of SNVs for each clone (**B,D,F,H,J,L)**, shuffled as described in Figure **1F**). **M-O** Box and whisker plots indicating percentage of SNVs within either a 500bp window of promoters (**M**), or a 5bp window of enhancers (**N**) or open chromatin (**O,** significant ATAC-seq peaks, which were identified in both replicates), grouped by parental clones (PAR) and SMARCA4 KO clones (KO), with clones plotted as individual points. **P-U** Box and whisker plots of SNVs that are not at potential covariate sites, with either PQS (**P,Q,R**, within a 5bp window) or G4 Quadron sites (**S,T,U**, within a 5bp window). Covariate sites, which overlapped with SNVs and were excluded from this analysis, were defined as either open chromatin (**P,S**, SNVs within a 500bp window of ATAC-seq peaks, which were identified in both replicates), regulatory elements ((REs), **Q,T,** SNVs within either 500bp of promoters or enhancers), or active histone PTMs (**R,U**, SNVs within 500bp of either H3K27ac, H3K4me1, H3K4me2, H3K4me3 or H3K9ac significant peaks. For **A-U**, statistical significance was assessed using a Mann Whitney test, error bars indicate min to max values, and all replicates are shown as dots.

Fig S6

A

		Total	DSBs +/-5bp SMARCA4 peaks
DSBs +/-5bp PQS	PAR_1	4,398	38
	PAR_2	5,863	55
	KO1_1	5,505	77
	KO1_2	6,626	75
	KO2_1	6,833	96
	KO2_2	10,658	127
DSBs NOT +/-5bp PQS	PAR_1	203,218	2,007
	PAR_2	274,358	2,321
	KO1_1	233,301	2,389
	KO1_2	295,906	2,919
	KO2_1	275,041	2,907
	KO2_2	446,341	3,887

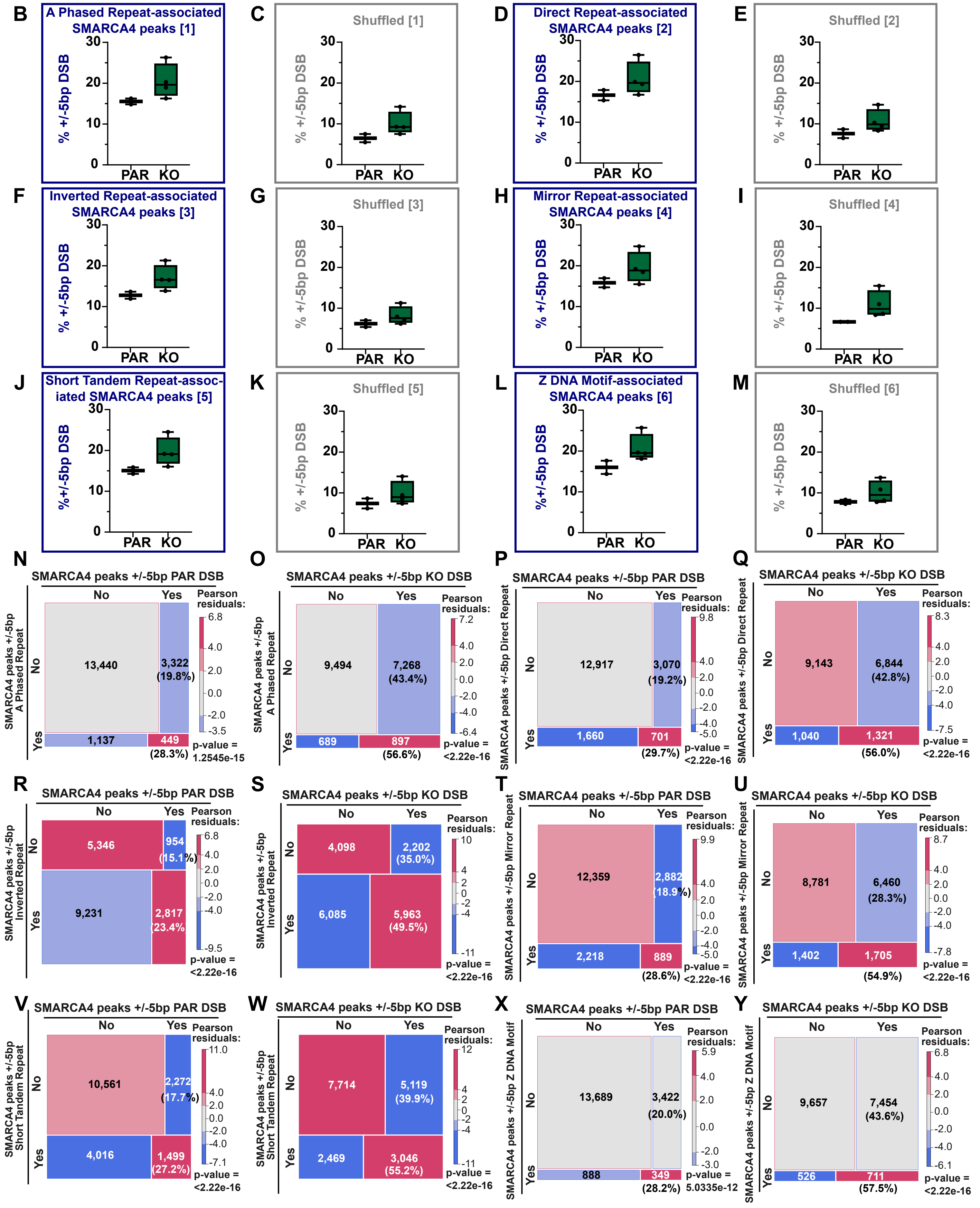

Fig S6. Non-B DNA-associated SMARCA4 binding sites are more likely to have DSBs, in absence of SMARCA4.

Table containing the total number of DSBs, either within a 5bp window of PQS sites or not, for each replicate, and of these, the number that are also within 5bp of consensus SMARCA4 peaks (significant in at least 2 replicates). **B-M** Box and whisker plots indicating percentage of Non-B DNA associated SMARCA4 peak groups that are within a 5bp window of DSBs, grouped by parental clone DSBs (PAR) and SMARCA4 KO clone DSBs (KO). SMARCA4 peak groups are defined as those that are within a 5bp window of predicted Non-B DNA sites (**B,D,F**,**H,J,L**), or shuffled controls, where coordinates of SMARCA4 peaks in **B,D,F**,**H,J,L** are shuffled around the genome respectively for **C,E,G,I,K,M**. **N-Y** Mosaic plots of observed frequencies of cooccurrence of SMARCA4 peaks, which are within a 5bp window of Non-B DNA sites, with either PAR DSBs (**N,P,R,T,V,X**) or SMARCA4 KO DSBs (**O,Q,S,U,W,Y**). Numbers of peaks in each subset are annotated, with percentages of peaks in each row that cooccur with DSBs. Tile sizes are proportional to the observed frequencies. Red indicates positive and blue indicates negative Pearson residuals from chi-square test. P-value indicates evidence for significant difference from expected values.

Fig S7

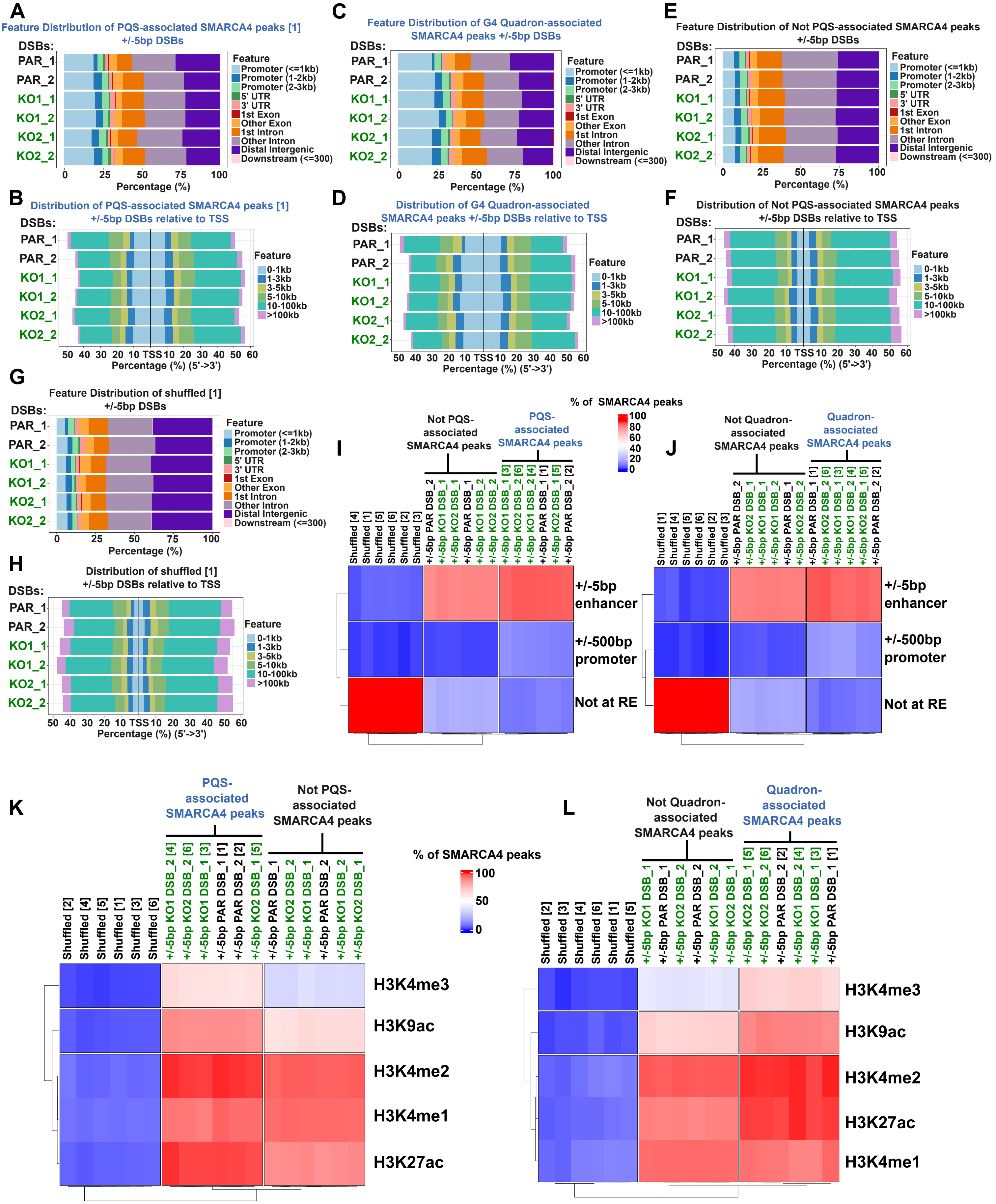

A
Feature Distribution of PQS-associated SMARCA4 peaks [1] +/-5bp DSBs
DSBs:
PAR_1
PAR_2
KO1_1
KO1_2
KO2_1
KO2_2
Feature
Promoter (<=1kb)
Promoter (1-2kb)
Promoter (2-3kb)
5' UTR
3' UTR
1st Exon
Other Exon
1st Intron
Other Intron
Distal Intergenic
Downstream (<=300)
0 25 50 75 100
Percentage (%)
B
Distribution of PQS-associated SMARCA4 peaks [1] +/-5bp DSBs relative to TSS
Feature
0-1kb
1-3kb
3-5kb
5-10kb
10-100kb
>100kb
50 40 30 20 10 TSS 10 20 30 40 50 60
Percentage (%) (5'->3')
C
Feature Distribution of G4 Quadron-associated SMARCA4 peaks +/-5bp DSBs
D
Distribution of G4 Quadron-associated SMARCA4 peaks +/-5bp DSBs relative to TSS
E
Feature Distribution of Not PQS-associated SMARCA4 peaks +/-5bp DSBs
F
Distribution of Not PQS-associated SMARCA4 peaks +/-5bp DSBs relative to TSS
G
Feature Distribution of shuffled [1] +/-5bp DSBs
H
Distribution of shuffled [1] +/-5bp DSBs relative to TSS
I
% of SMARCA4 peaks
100 80 60 40 20 0
Not PQS-associated SMARCA4 peaks
PQS-associated SMARCA4 peaks
Shuffled [4]
Shuffled [1]
Shuffled [5]
Shuffled [6]
Shuffled [2]
Shuffled [3]
+/-5bp PAR DSB_2
+/-5bp KO1 DSB_1
+/-5bp KO2 DSB_1
+/-5bp PAR DSB_1
+/-5bp KO1 DSB_2
+/-5bp KO2 DSB_2
+/-5bp KO1 DSB_1 [3]
+/-5bp KO2 DSB_2 [6]
+/-5bp KO1 DSB_2 [4]
+/-5bp PAR DSB_1 [1]
+/-5bp KO2 DSB_1 [5]
+/-5bp PAR DSB_2 [2]
+/-5bp enhancer
+/-500bp promoter
Not at RE
J
Not Quadron-associated SMARCA4 peaks
Quadron-associated SMARCA4 peaks
Shuffled [1]
Shuffled [4]
Shuffled [5]
Shuffled [6]
Shuffled [2]
Shuffled [3]
+/-5bp PAR DSB_2
+/-5bp KO2 DSB_1
+/-5bp KO1 DSB_1
+/-5bp KO1 DSB_2
+/-5bp PAR DSB_1
+/-5bp KO2 DSB_2
+/-5bp PAR DSB_1 [1]
+/-5bp KO2 DSB_2 [6]
+/-5bp KO1 DSB_1 [3]
+/-5bp KO1 DSB_2 [4]
+/-5bp KO2 DSB_1 [5]
+/-5bp PAR DSB_2 [2]
+/-5bp enhancer
+/-500bp promoter
Not at RE
K
PQS-associated SMARCA4 peaks
Not PQS-associated SMARCA4 peaks
% of SMARCA4 peaks
100 50 0
Shuffled [2]
Shuffled [4]
Shuffled [5]
Shuffled [1]
Shuffled [3]
Shuffled [6]
+/-5bp KO1 DSB_2 [4]
+/-5bp KO2 DSB_2 [6]
+/-5bp KO1 DSB_1 [3]
+/-5bp PAR DSB_1 [1]
+/-5bp PAR DSB_2 [2]
+/-5bp KO2 DSB_1 [5]
+/-5bp PAR DSB_1
+/-5bp KO2 DSB_2
+/-5bp KO1 DSB_1
+/-5bp PAR DSB_2
+/-5bp KO1 DSB_2
+/-5bp KO2 DSB_1
H3K4me3
H3K9ac
H3K4me2
H3K4me1
H3K27ac
L
Not Quadron-associated SMARCA4 peaks
Quadron-associated SMARCA4 peaks
Shuffled [2]
Shuffled [3]
Shuffled [4]
Shuffled [6]
Shuffled [1]
Shuffled [5]
+/-5bp KO1 DSB_1
+/-5bp PAR DSB_1
+/-5bp KO2 DSB_2
+/-5bp PAR DSB_2
+/-5bp KO1 DSB_2
+/-5bp KO2 DSB_1
+/-5bp KO2 DSB_1 [5]
+/-5bp KO2 DSB_2 [6]
+/-5bp PAR DSB_2 [2]
+/-5bp KO1 DSB_2 [4]
+/-5bp KO1 DSB_1 [3]
+/-5bp PAR DSB_1 [1]
H3K4me3
H3K9ac
H3K4me2
H3K27ac
H3K4me1

Fig S7. G4- and DSB-associated SMARCA4 peaks are more likely to occur at regulatory elements.

A-H Stacked colour bar charts representing the genomic distribution of either PQS-associated SMARCA4 peaks (within a 5bp window of PQS) that are also within 5bp of a DSB (**A,B**), Quadron-associated SMARCA4 peaks (within a 5bp window of Quadron) that are also within 5bp of a DSB (**C,D**), or Not PQS-associated SMARCA4 peaks (not within a 5bp window of PQS) that are within 5bp of a DSB (**E,F**), or a shuffled control, where the coordinates of G4-associated SMARCA4 peaks that are also within 5bp of a DSB have been shuffled around the genome (**G,H**), shown separately for each replicate of INDUCE-seq for parental (labelled in black) and SMARCA4 KO clones (labelled in green). Stacked colour bar charts represent either feature categories (**A,C,E,G**) or relative distance to TSS (**B,D,F,H**). **I-J** Hierarchical heatmaps representing the percentage of SMARCA4 peaks within 500bp of promoters, 5bp of enhancers or neither (Not at RE (regulatory element)). **K-L** Heatmap representing the percentage of SMARCA4 peaks within 5bp window of significant histone PTM peaks. For **I-L**, SMARCA4 peaks are defined as either PQS-associated that are also within 5bp of DSBs (**I,K**) or G4 Quadron-associated that are also within 5bp of DSBs (**J,L**) (labelled in blue), those that are either Not PQS--associated but that are within 5bp of DSBs (**I,K**) or Not G4 Quadron-associated but that are within 5bp of DSBs (**J,L**), and for all, shuffled controls where the coordinates of these peak groups (specified in square brackets) are shuffled around the genome.

Fig S8

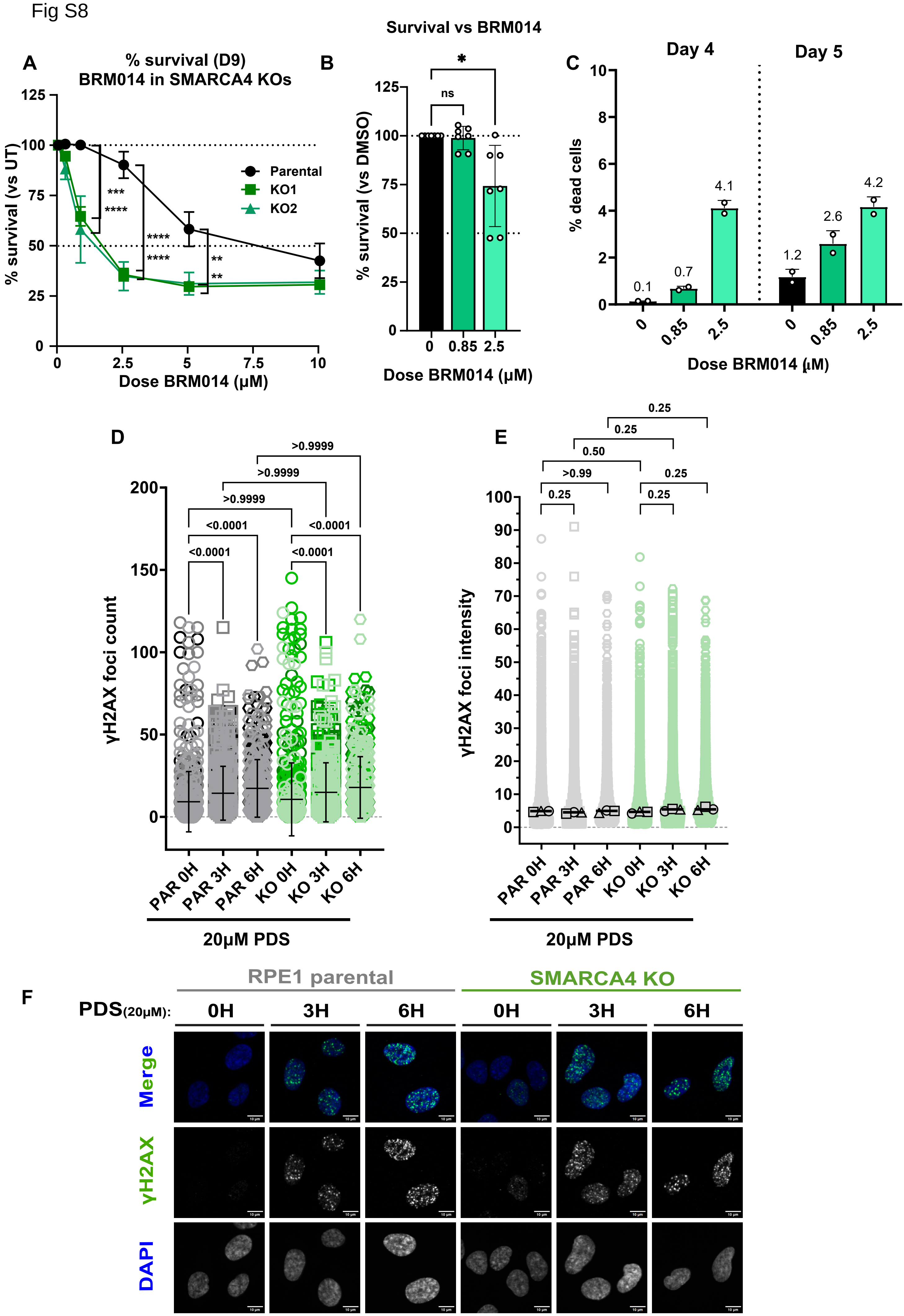
A
% survival (D9)
BRM014 in SMARCA4 KOs
% survival (vs UT)
Parental
KO1
KO2
***
****
****
****
**
**
Dose BRM014 (μM)
B
Survival vs BRM014
*
ns
% survival (vs DMSO)
Dose BRM014 (μM)
C
Day 4
Day 5
% dead cells
0.1
0.7
4.1
1.2
2.6
4.2
Dose BRM014 (μM)
D
γH2AX foci count
<0.0001
<0.0001
<0.0001
<0.0001
<0.0001
>0.9999
>0.9999
>0.9999
PAR 0H
PAR 3H
PAR 6H
KO 0H
KO 3H
KO 6H
20μM PDS
E
γH2AX foci intensity
0.25
0.25
0.25
0.25
0.50
>0.99
0.25
PAR 0H
PAR 3H
PAR 6H
KO 0H
KO 3H
KO 6H
20μM PDS
F
RPE1 parental
SMARCA4 KO
PDS(20μM):
0H
3H
6H
0H
3H
6H
Merge
γH2AX
DAPI

Fig S8. SMARCA4 knockout cells are sensitive to the ATPase inhibitor BRM014

A SRB % survival of BRM014 in SMARCA4 KOs, after 9 days of culture (D9). Data were analysed by two-way ANOVA with Tukey's multiple comparisons test. $^{**}p<0.005$, $^{***}p<0.0005$, $^{****}<p<0.0001$, n=3, mean±SEM. **B** Bar graph showing percentage of survival of parental RPE1 following treatment with either 0.85 or 2.5 μM BRM014 or DMSO vehicle control, for 6 days of culture. Points correspond to independent biological replicates, n = 7, mean ± SEM, data were analysed by ordinary one-way ANOVA with Kruskal-Wallis test. $^{*}p<0.05$. **C** Quantification of the % of dead cells, defined as the % of propidium-iodide positive cells in a cell population, after 4 (left) or 5 (right) days in culture, in RPE1 parental cells with two doses of ATPase inhibitor BRM014 (0.85, 2.5 μM), or DMSO vehicle control (0). n=2, mean±SEM. **D** Quantification of γH2AX foci in RPE1 parental or SMARCA4 knockout cells under unperturbed or PDS treatment (20 μM, 3H (hours) and 6H). Error bars indicate mean ± SD, and statistical significance was assessed using a Kruskal-Wallis test with multiple comparisons. **E** Quantification of γH2AX foci intensity per cell in RPE1 parental or SMARCA4 knockout cells under unperturbed or PDS treatment (20 μM, 3H and 6H). Coloured points represent individual foci, and shaped points indicate the different biological replicate. The horizontal line represents the median across replicates. Statistical analysis was performed using a one-sample Wilcoxon test of the medians. **F** Representative images of γH2AX immunofluorescence, as quantified in Additional file 1: Fig S7D-E are shown. Scale bar represents 10 μm.

Fig S9

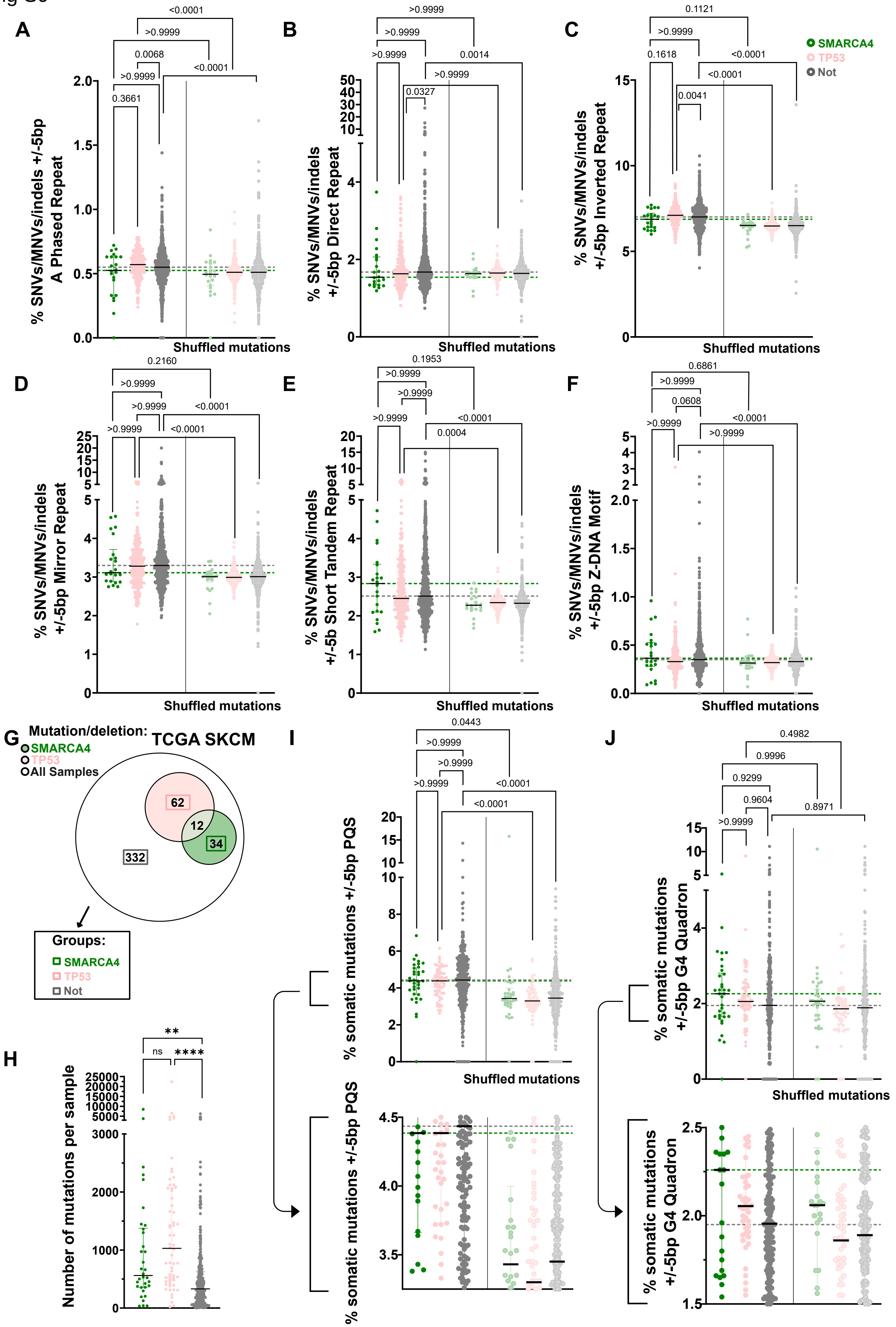
A
B
C
D
E
F
G
H
I
J
% SNVs/MNVs/indels +/-5bp A Phased Repeat
% SNVs/MNVs/indels +/-5bp Direct Repeat
% SNVs/MNVs/indels +/-5bp Inverted Repeat
% SNVs/MNVs/indels +/-5bp Mirror Repeat
% SNVs/MNVs/indels +/-5b Short Tandem Repeat
% SNVs/MNVs/indels +/-5bp Z-DNA Motif
Shuffled mutations
SMARCA4
TP53
Not
Mutation/deletion:
TCGA SKCM
All Samples
62
12
34
332
Groups:
SMARCA4
TP53
Not
Number of mutations per sample
**
ns
****
% somatic mutations +/-5bp PQS
% somatic mutations +/-5bp G4 Quadron

Fig S9. SMARCA4 mutant samples more likely to have mutations near predicted G4s and Short Tandem Repeats.

A-F Scatter plots indicating percentages of SNVs/MNVs/indels within a 5bp window of predicted Non-B DNA sites in PCAWG samples, grouped by mutation status (as defined in Figure **7A**), with each point representing an individual patient sample and a line for the median. Dotted lines are plotted for the median percentage of the SMARCA4 mutant samples (green), and the samples with Not SMARCA4 or TP53 mutation/deletion (grey). **G** Venn diagram indicating the overlap of TCGA SKCM patient samples (white) with either mutation or deletion of SMARCA4 (green) or TP53 (pink). Patient sample groups are defined as in the box, with boxes around the numbers in the Venn diagram that correspond to the number of samples in each group. **H** The number of mutations per sample in TCGA SKCM patients, separated by mutation status, with each sample represented as an individual point, and a line at the median. **I-J** Scatter plots indicating percentages of somatic mutations within a 5bp window of PQS sites **(I)** or G4-Quadron sites **(J)** in TCGA SKCM samples, grouped by mutation status (as defined in **G**), with each point representing an individual patient sample and a line for the median (top panel), with a zoomed inset (bottom panel). Dotted lines are plotted for the median percentage of the SMARCA4 mutant samples (green), and the samples with Not SMARCA4 or TP53 mutation/deletion (grey). For **A-F** and **H-J**, statistical significance was assessed using a Kruskal-Wallis test with Dunn's multiple comparisons test (for **H**, **: <0.005 and ****: <0.0001).

Fig S10

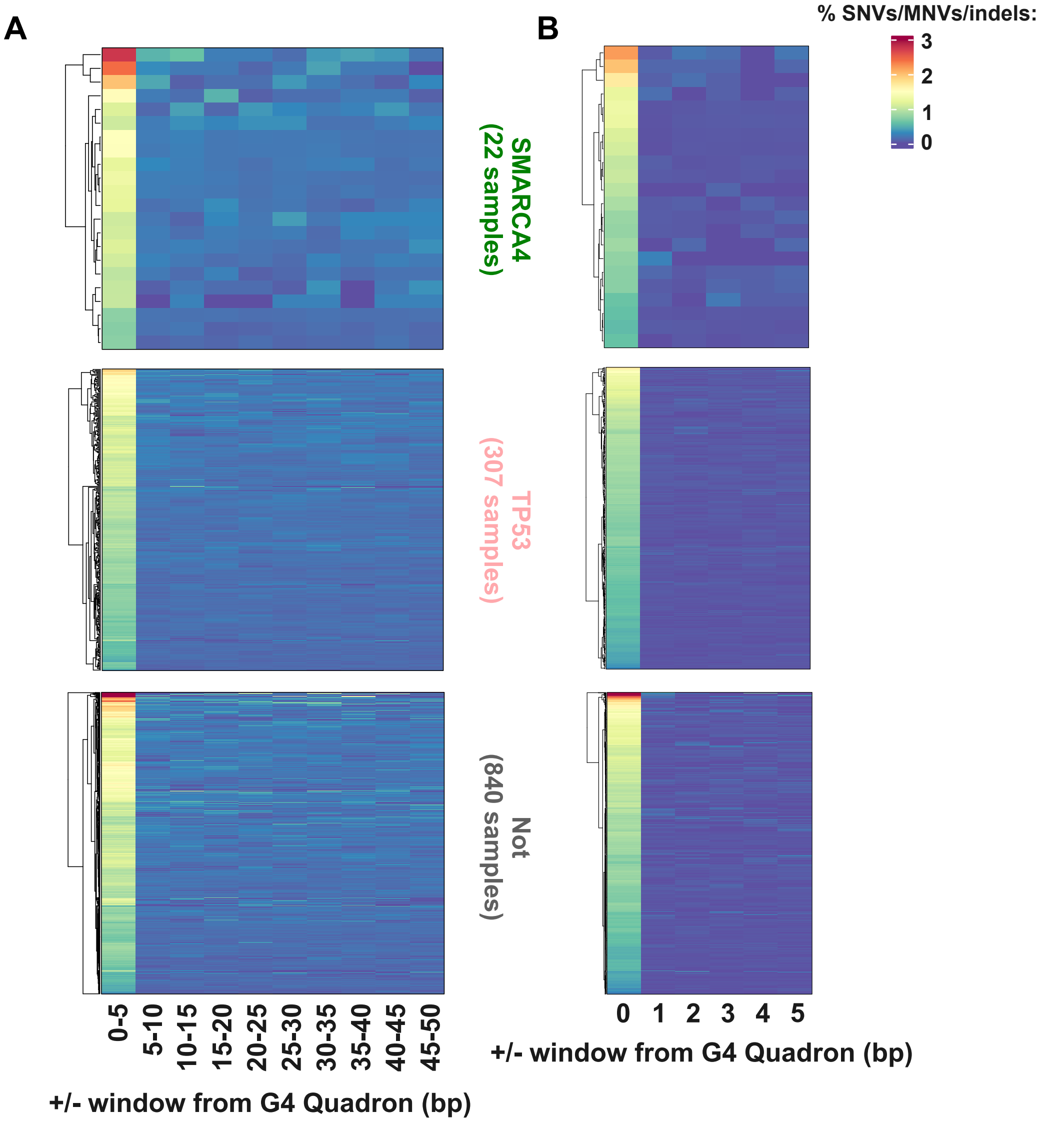

A
B
% SNVs/MNVs/indels:
3
2
1
0
SMARCA4
(22 samples)
TP53
(307 samples)
Not
(840 samples)
0-5
5-10
10-15
15-20
20-25
25-30
30-35
35-40
40-45
45-50
+/- window from G4 Quadron (bp)
0
1
2
3
4
5
+/- window from G4 Quadron (bp)

Fig S10. SMARCA4 mutant samples are more likely to have mutations near G4 Quadron sites.

A-B Hierarchical heatmaps representing the percentage of SNVs/MNVs/indels that are within a window of G4 Quadron sites, either shown in 5bp bins from 0-50bp (**A**) or in 1bp bins from 0-5bp (**B**) for PCAWG patient samples labelled and plotted separately for each patient group.
