# Supplementary material for: The SMARCA4 subunit of the SWI/SNF complex prevents genome instability at G quadruplexes
Source: Genome Biol. 2026 Apr 20;27:180. doi: 10.1186/s13059-026-04080-4 (PMC13224610; doi:10.1186/s13059-026-04080-4)
Supplement: Supplementary file 2 — Additional file 2: Figs. S1 – S5. Original, uncropped images of all gels and blots. [file 13059_2026_4080_MOESM2_ESM.pdf]

Fig S1

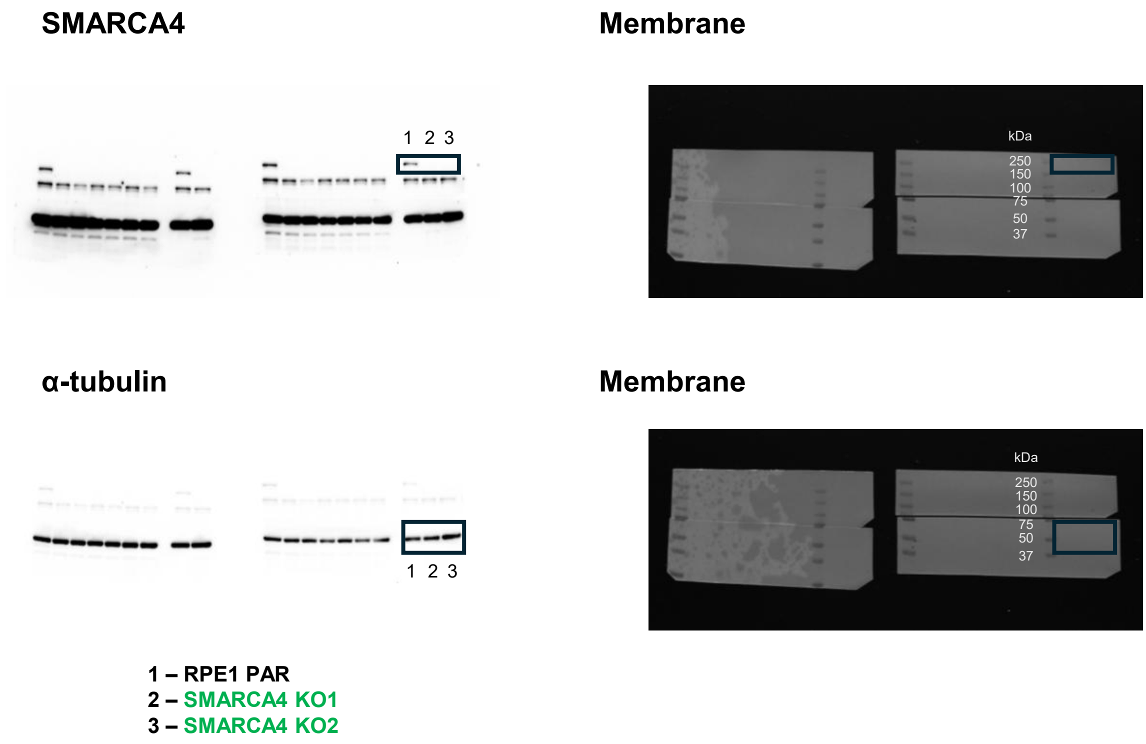
SMARCA4
Membrane
1 2 3
kDa
250
150
100
75
50
37
α-tubulin
Membrane
1 2 3
kDa
250
150
100
75
50
37
1 – RPE1 PAR
2 – SMARCA4 KO1
3 – SMARCA4 KO2

Fig S1. Raw Western blots from Fig S2A.

Full-length, uncropped scans of Western blots from Additional file 1: Fig S2A, including all edges and molecular weight markers, labelled consistently to the related main figure panel and with the cropped regions indicated.

Fig S2

SMARCA4

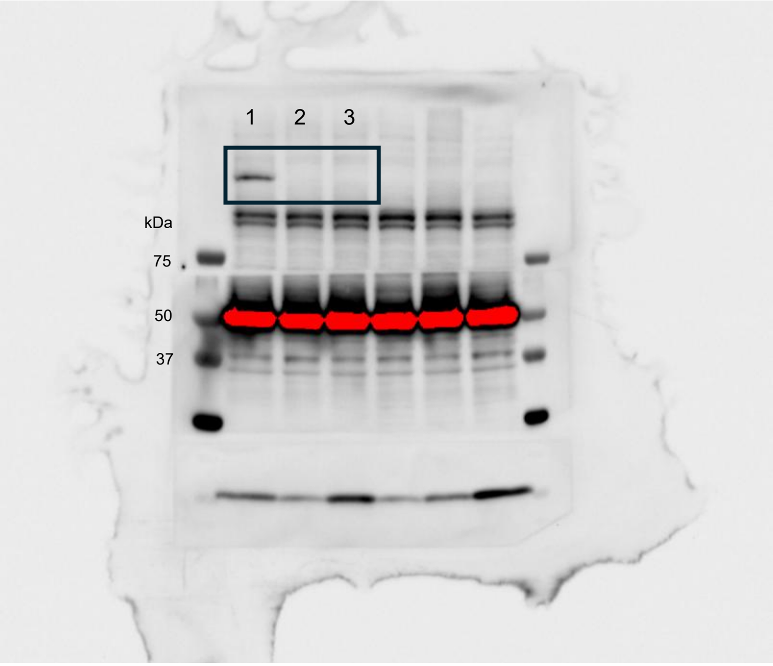

α-tubulin

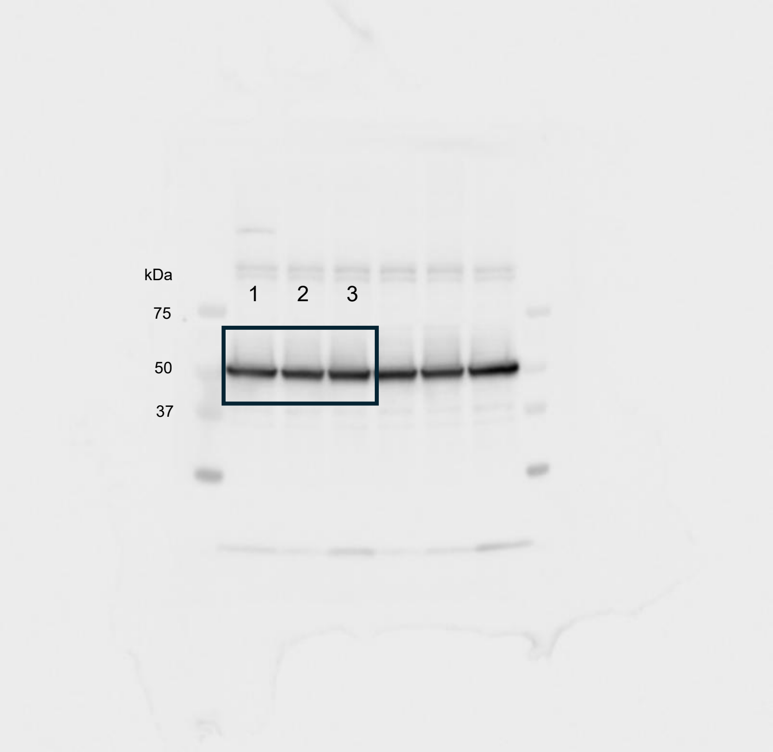

1 – RPE1 PAR
2 – SMARCA4 KO3
3 – SMARCA4 KO1

Fig S2. Raw Western blots from Fig S4A.

Full-length, uncropped scans of Western blots from Additional file 1: Fig S4A, including all edges and molecular weight markers, labelled consistently to the related main figure panel and with the cropped regions indicated.

Fig S3

SMARCA4

PAR clones

1 2 3

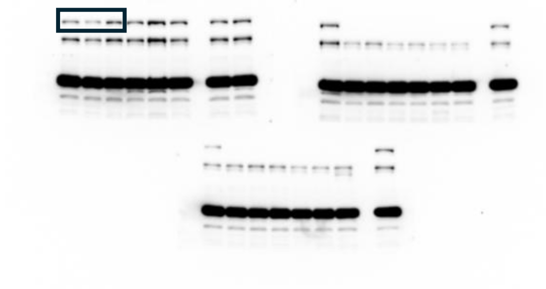

Membrane

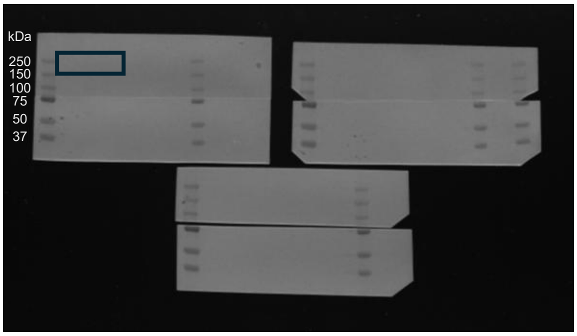

α-tubulin

PAR clones

1 2 3

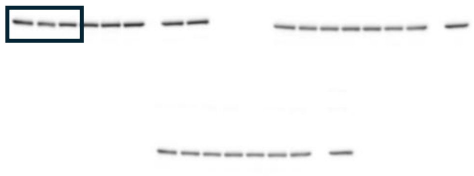

Membrane

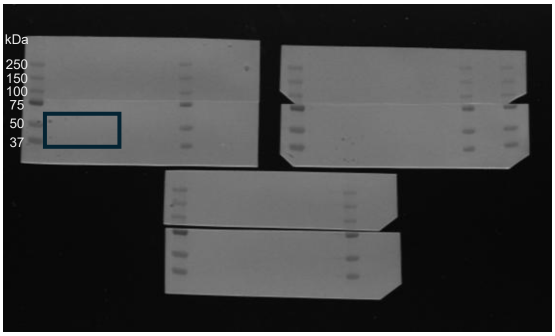

Fig S3. Raw Western blots from Fig S4B.

Full-length, uncropped scans of Western blots from Additional file 1: Fig S4B, including all edges and molecular weight markers, labelled consistently to the related main figure panel and with the cropped regions indicated.

Fig S4

SMARCA4

Membrane

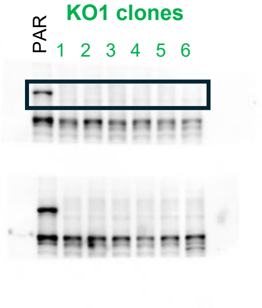

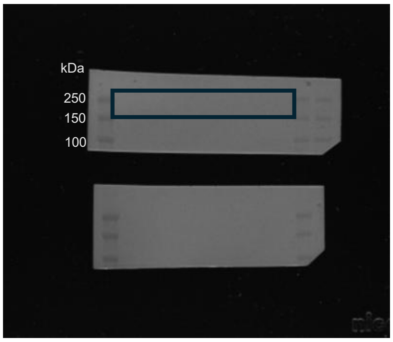

α-tubulin

Membrane

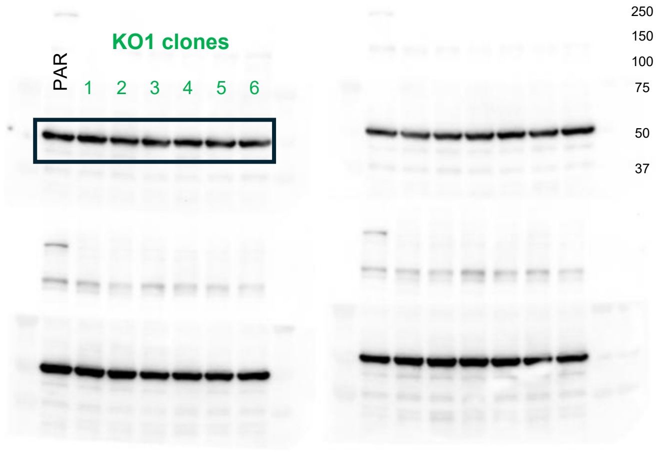

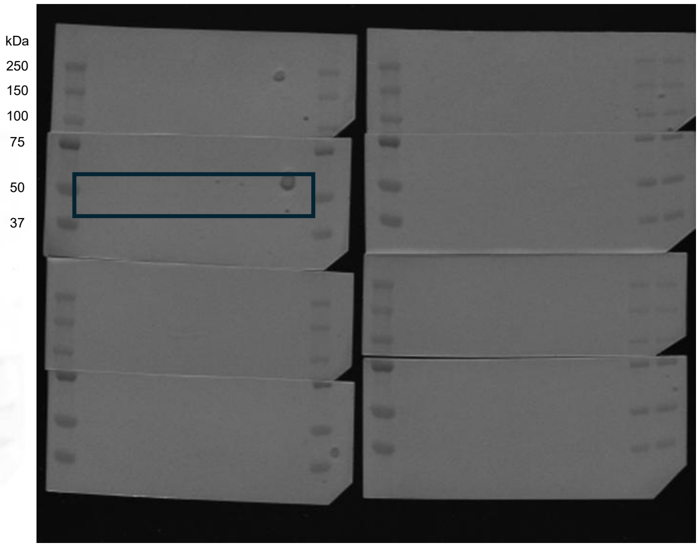

Fig S4. Raw Western blots from Fig S4C.

Full-length, uncropped scans of Western blots from Additional file 1: Fig S4C, including all edges and molecular weight markers, labelled consistently to the related main figure panel and with the cropped regions indicated.

Fig S5

SMARCA4

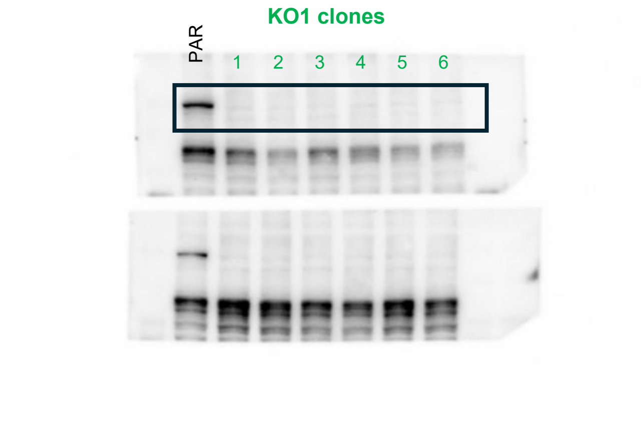

Membrane

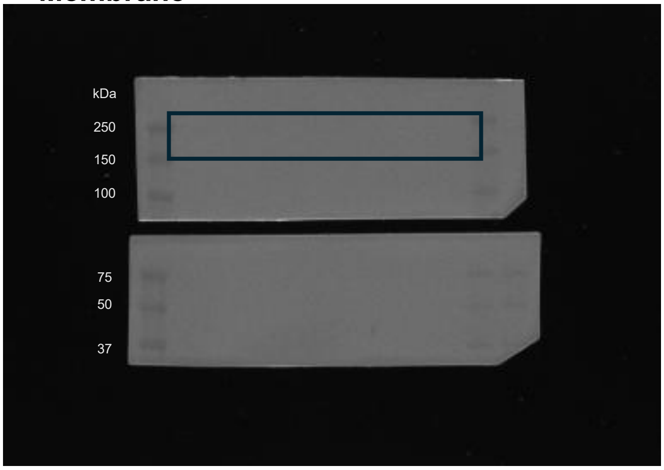

α-tubulin

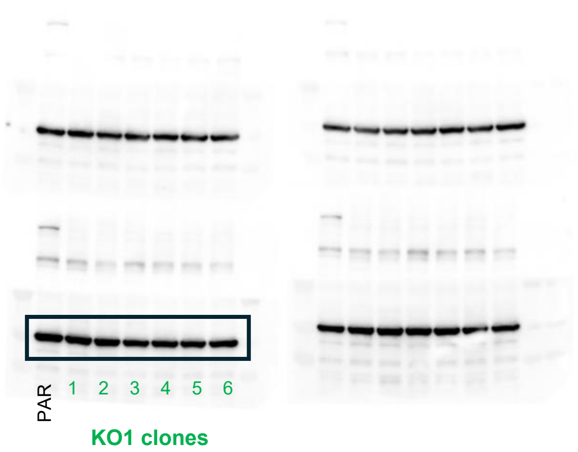

Membrane

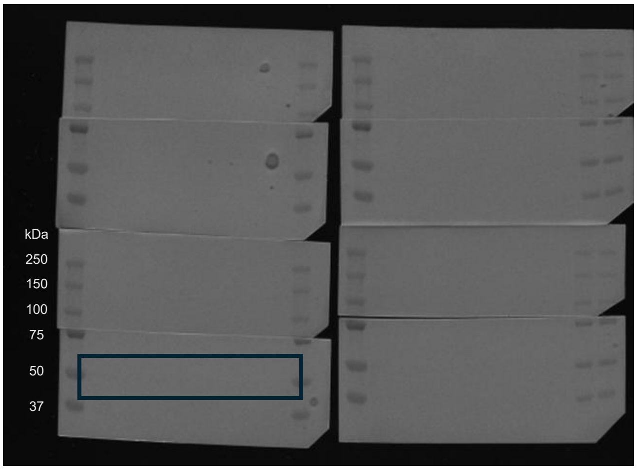

Fig S5. Raw Western blots from Fig S4D.

Full-length, uncropped scans of Western blots from Additional file 1: Fig S4D, including all edges and molecular weight markers, labelled consistently to the related main figure panel and with the cropped regions indicated.
